# Tailoring Conversion‐Reaction‐Induced Alloy Interlayer for Dendrite‐Free Sulfide‐Based All‐Solid‐State Lithium‐Metal Battery

**DOI:** 10.1002/advs.202300985

**Published:** 2023-04-21

**Authors:** Yuhao Liang, Chen Shen, Hong Liu, Chao Wang, Dabing Li, Xiaoxue Zhao, Li‐Zhen Fan

**Affiliations:** ^1^ Beijing Advanced Innovation Center for Materials Genome Engineering Beijing Key Laboratory for Advanced Energy Materials and Technologies University of Science and Technology Beijing Beijing 100083 China; ^2^ Institute of Materials Science Technical University of Darmstadt 64 287 Darmstadt Germany

**Keywords:** alloy interlayer, all‐solid‐state batteries, conversion reaction, lithium metal anodes, sulfide solid electrolytes

## Abstract

Utilization of lithium (Li) metal anodes in all‐solid‐state batteries employing sulfide solid electrolytes is hindered by diffusion‐related dendrite growth at high rates of charge. Engineering ex‐situ Li‐intermetallic interlayers derived from a facile solution‐based conversion‐alloy reaction is attractive for bypassing the Li0 self‐diffusion restriction. However, no correlation is established between the properties of conversion‐reaction‐induced (CRI) interlayers and the deposition behavior of Li0 in all‐solid‐state lithium‐metal batteries (ASSLBs). Herein, using a control set of electrochemical characterization experiments with LixAgy as the interlayer in different battery chemistries, this work identifies that dendritic tolerance in ASSLBs is susceptible to the surface roughness and electronic conductivity of the CRI‐alloy interlayer. This work thereby tailors the CRI‐alloy interlayer from the typical mosaic structure to a hierarchical gradient structure by adjusting the pit corrosion kinetics from the (de)solvation mechanism to an adsorption model, yielding a smooth organic‐rich outer layer and a composition‐regulated inorganic‐rich inner layer composed mainly of lithiophilic LixAgy and electron‐insulating LiF. Ultimately, desirable roughness, conductivity, and diffusivity are integrated simultaneously into the tailored CRI‐alloy interlayer, resulting in dendrite‐free and dense Li deposition beneath the interlayer capable of improving battery cycling stability. This work provides a rational protocol for the CRI‐alloy interlayer specialized for ASSLBs.

## Graphical abstract

The correlations between the properties of prevalent conversion‐reaction‐induced (CRI) alloy interlayers and dendrite growth in all‐solid‐state lithium‐metal batteries (ASSLBs) are established in this work. A tailored CRI alloy interlayer based on the adsorption‐alloy mechanism is thereby proposed to achieve dendrite‐free and dense Li^0^ deposition capable of prolonged battery cycling stability at high rates of charge.

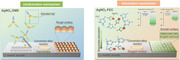

## 1 Introduction

On the cusp of the decarbonization revolution, technological breakthroughs in energy‐dense and safe batteries are becoming increasingly pivotal.^[^ ^1^ ^]^ In this respect, all‐solid‐state lithium‐metal batteries (ASSLBs) that integrate nonflammable solid electrolytes (SEs) in conjunction with high‐specific‐energy lithium (Li) metal anode hold promise for a paradigm shift in post‐lithium‐ion batteries.^[^ ^2^ ^]^ With the recent discovery of SE materials, the practical deployment of ASSLBs has become much closer to reality. In particular, sulfide‐type SEs (e.g., argyrodite Li_6_PS_5_Cl, Li_10_GeP_2_S_12_, Li_3_PS_4_, and Li_7_P_3_S_11_) benefit from the combined ultra‐high ionic conductivities (>1 mS cm^−1^) and favorable mechanical deformability and appear to be a possible near‐future SE. In addition, sufficiently high shear modulus and Li^+^ transference number of ceramic SEs are initially expected to physically curb the penetration of Li dendrites and allow uniform Li deposition, thus permitting the utilization of Li‐metal anodes to realize potentially higher energy density.^[^ ^3^ ^]^

Unfortunately, premature cell shorting in ASSLBs is increasingly observed even at much low current densities (e.g., 200 µA cm^−2^) compared to cells employing liquid electrolytes (LEs). Various macroscopic measurements corroborated severe dendrite penetration into SE separators via grain boundaries and pores.^[^ ^4^ ^]^ It is generally acknowledged that electron leakage and existing flaws of bulk SEs are the primary causes of counterintuitive dendrite growth inside SE separators.^[^ ^5^ ^]^ Meanwhile, once sulfide SEs and Li anodes are in contact, parasitic reactions occur at the anode interface due to their unmatched energy gaps. Incompatible reactions render the dynamical evolution of interphases yielding significant interfacial resistance, thereby impairing dendrite growth tolerance.^[^ ^6^ ^]^ Concerning initial dendrite nucleation, a consensus steered by scientific research is that the interfacial properties between the Li anode and the SE separator play a crucial role in regulating Li deposition and subsequent dissolution. On the one hand, poor wettability of SEs toward planar Li anodes leads to limited solid‐solid contact and inhomogeneous mass transport across the interface, which creates hotpots for preferred dendrite nucleation and proliferation.^[^ ^7^ ^]^ On the other hand, solid electrolyte interphases (SEI; e.g., Li_2_S and Li_3_P) generated by the reductive degradation of sulfide SEs cannot block dendrite propagation effectively because of their high electronic conductivity, low interface energy against Li, and inhomogeneity in both morphology and composition.^[^ ^8^ ^]^ As a result of uneven Li deposition at the interface, pressure builds up locally at the hotpots, and current‐induced intragranular fractures initiate and propagate throughout the SE separator with the filling of Li^0^, incurring a risk of short circuits.

In this regard, based on prevalent interface engineering concepts, the insertion of protective interlayers such as polymers [propylene carbonate, poly(ethylene oxide), alucone], inorganics (LiH_2_PO_4_, LiF, LiI, and LiPON) or quasi‐solid modified layers as a stable artificial SEI or interlayer buffer can mitigate adverse reactions, steer ionic flux and regulate current distribution, and thereby homogenize Li deposition.^[^ ^9^ ^]^ Although these approaches effectively reduce interfacial resistance via diverse mechanisms, the critical current density (CCD) in most studies is still too low for practical applications. It turns out that under the operation with high current densities, the Li migration rate at the SE/Li interface exceeds the Li replenishment rate by Li^0^ diffusion or deformation, resulting in the accumulation of Li vacancies that evolve into nonhomogeneous voids.^[^ ^10^ ^]^ These voids cause uneven current contraction locally and a high local hydrostatic stress, triggering the site‐specific nucleation and growth of dendrites at the hotpots based on the (electro)chemomechanics mechanism.

To overcome the limitation of Li^0^ self‐diffusivity, embedding Li‐rich‐intermetallic phases into the engineered interface can possibly extend Li replenishing from vacancy diffusion to chemical diffusion.^[^ ^11^ ^]^ Often, lithiophilic alloy interface design has been certified as the strategy of choice for multiple electrolyte systems in combination with Li anodes. Noteworthily, Nazar et al. proposed a solution‐based surface chemistry route to form a series of protective interlayers comprised of Li*_x_* M*_y_* (M can be In, Zn, Bi, As) compounds chemically bound onto Li foil via direct reduction of metal chlorides by Li^0^ followed by self‐alloying. This solution‐based pretreatment methodology is facile and inexpensive to stabilize Li anodes compared to other complex film‐forming procedures (e.g., atomic layer deposition, pulsed laser deposition, and sputter deposition).^[^ ^12^ ^]^ Inspired by this paradigm, a variety of high‐Li‐affinity interlayers, derived from Li‐alloyable metal compounds (e.g., SnF_2_, SbF_3,_ ZnO, SnTFSI, GeCl_4_, and Ag_2_S) that have a conversion‐alloy reaction with Li^0^, were widely employed to lower the Li nucleation overpotential and homogenize Li deposition in LE‐based Li‐metal batteries.^[^ ^13^ ^]^ Inheriting the existing know‐how and wisdom of dendrite suppression from liquid lithium‐ion batteries and applying it to ASSLBs is a quick way out. Nonetheless, considering that the attributed factors to dendrite growth in ASSLBs exhibit discrepancies compared with that in LE systems, the conversion‐reaction‐induced (CRI) alloy interlayer applied in sulfide‐based SE systems seem to be stuck in a new bottleneck since i) most alloy interlayers are electronic conductors that accelerate electron escape to the SE separator and promote charge transfer reactions to take place right between the interlayer and the SE separator or inside the SE separator; ii) rapid pit corrosion kinetics result in the surface inhomogeneity of re‐engineered Li electrodes with randomly distributed pits and protrusions for preferential dendrite growth. The elucidation of CRI‐alloy interlayers as an effective Li diffusion medium and their corresponding design principle in ASSLBs are not well established yet. Understanding the origins of dendrite growth in ASSLBs with CRI‐alloy interlayers and exploring possible strategies to mitigate it is therefore crucial for the practical realization of ASSLBs.

By means of a control set of electrochemical characterization experiments, soaking Li electrodes briefly into a solution of AgNO_3_ in dimethyl ether (DME) to *ex‐situ* construct CRI‐alloy interlayers, we show that dendrite growth tolerance in ASSLBs is susceptible to the morphology, structure, and composition of the interlayer. We recognize that the effect of surface roughness of Li electrodes pretreated by the surface chemistry method on the local current contraction associated with dendritic growth at the interface will be amplified in ASSLBs due to the absence of liquid wetting. In addition, electron leakage from the CRI‐alloy interlayer to the SE separator triggers the propensity for the electric breakdown of sulfide SEs that further upsets the uniform Li plating/stripping.

Whereafter, by replacing DME with selected fluoroethylene carbonate (FEC) solvent, we adjusted the conversion reaction kinetics from the typical (de)solvation mechanism to an adsorption model to tailor the composition and surface homogeneity of the CRI‐alloy layer. The tailored CRI‐alloy layer presented a hierarchical gradient structure with a smooth organic‐rich outer layer and a composition‐regulated inorganic‐rich inner layer composed mainly of ion‐conducting Li*_x_* Ag*_y_* and electron‐insulating LiF. Comprehensive material characterization, electrochemical performance evaluation, and simulation confirmed that the defect‐free surface of the re‐engineered electrode inhibits preferential dendrite nucleation and growth at artificial hotpots. Meanwhile, the synergy of Li*_x_* Ag*_y_* with a low Li diffusion barrier and LiF with electron‐blocking properties enables dendrite‐free and dense Li deposition beneath the CRI‐alloy interlayer, which maintains a stable and intact interface during repeated Li plating/stripping. As the tailored CRI‐alloy interlayer colligated both low surface roughness, low electronic conductivity, and low Li diffusion barrier, an ultrahigh CCD of 1.7 mA cm^−2^ was realized in Li‐Li symmetric cells, and superior cycling stability of 82.7% over 300 cycles at 0.3 C was demonstrated in ASSLBs.

## 2 Results and Discussion

### 2.1 Evaluation of Routine CRI‐alloy Interlayer in SE System

In controlled experiments, AgNO_3_ was chosen as the representative Li surface treatment chemical to form a solid‐solution‐based Li‐Ag alloy that shows high structural stability during cycling, thus avoiding possible contact loss at the interface caused by lithium‐saturated intermetallic compounds (e.g., Li‐Al and Li‐Si alloy).^[^ ^14^ ^]^ The prototypical CRI‐alloy interlayer was initially constructed on the Li electrode surface by soaking the Li foil in a solution with typical DME as the solvent and AgNO_3_ as the solute (denoted henceforth as ANO‐DME) at room temperature (RT). The schematic diagram in Figure S1, Supporting Information, illustrates the simple treatment methodology toward the CRI‐alloy interlayer on the Li electrode surface, by which the AgNO_3_ solute undergoes a substitution reaction with Li^0^ to form LiNO_3_ and Ag products (Li + AgNO_3_ → Ag + LiNO_3_) and the substituted Ag products are then alloyed with the underlying Li^0^ until a Li‐rich Li‐Ag alloy is achieved (*x*Li + *y*Ag → Li_*x*_Ag_*y*_). Similar to those reported conversion reactions between metal compounds and Li^0^, this treatment process is thermodynamically spontaneous and fast, given the distinct electronegativity between metallic Li and Ag.^[^ ^15^ ^]^ The Li electrode color discernibly changes within seconds upon dipping it in the ANO‐DME solution (Figure S2, Supporting Information). After sequential conversion reaction and self‐alloying process, crystalline Li*_x_* Ag*_y_* is formed in the CRI‐alloy interlayer, as validated by X‐ray diffraction (XRD) measurement (Figure S3, Supporting Information). The absence of LiNO_3_ signal peaks is due to conversion reaction residues being washed away with pure DME.

Scanning electron microscopy (SEM) images of CRI‐alloy‐protected Li electrodes with varying concentrations of ANO‐DME (denoted as ANO*x*‐DME‐Li, where *x* can be 5, 10, 40, or 100 mm) in Figure S4, Supporting Information, reveal that the surface morphology and thickness of the CRI‐alloy interlayer are concentration‐ and time‐dependent in the reaction solution of ANO‐DME. Specifically, when the concentration of salt solute is too low (e.g., 5 mm), the CRI‐alloy layer cannot cover the entire Li electrode surface, while upon the reaction time is too long or the concentration is too high, a nonuniform and thick interlayer would frustrate the Li diffusion and upset lithium deposition. After optimized experiments, the low concentration of ANO‐DME (i.e., 10 and 40 mm) in conjunction with a moderate duration time (i.e., 20 s) renders a little rough and densely packed nanoparticles distributed on the Li foil surface.

To validate the dendrite suppression capability of the as‐formed CRI alloy interlayer, Li stripping/plating measurements of Li‐Li symmetric cells without and with the interlayer were carried out in both LE and SE systems. Figure S5, Supporting Information),shows the voltage profiles as a function of time of the symmetric cells in the ether‐based LE (current density: 1 mA cm^−2^, areal capacity: 1 mAh cm^−2^). The pristine Li was short‐circuited with 242 h due to dendrites, signaled by voltage fluctuations and large overpotentials. In contrast, both ANO10‐DME‐Li and ANO40‐DME‐Li assembled symmetric cells display smaller and relatively stable polarization over 1800 h without any sign of short‐circuiting. A comparison of the Nyquist plots of Li‐Li symmetric cells at the fresh condition and during cycling reveals faster charge transport through the CRI‐alloy interlayer than the native SEI formed on the pristine Li anode (Figure S6, Supporting Information). In addition, pristine Li undergoes side reactions with the LE, resulting in an increase in charge transfer resistance (*R*_ct_) during cycling, while CRI‐alloy‐protected anodes exhibit lower and almost constant resistance during repeated plating/stripping, implying the interlayer remains stable during cycling that ensures fast charge transfer or Li diffusivity within the layer. The visual Li deposition morphologies with the CRI‐alloy interlayer were characterized by SEM, as shown in Figure S7, Supporting Information. After initial electrodeposition with 1 mAh cm^−2^ of Li^0^, the pristine Li electrode surface became extremely rough, with many cracks caused by loosely arranged Li granules. These high‐surface‐area dendrites would aggravate parasitic reactions with the LE, causing the pulverization of deposits after repeated plating/stripping. Comparatively, the CRI alloy‐protected Li electrode shows non‐dendritic, dense, and seamless lithium deposition. The above results suggest an appropriate CRI‐alloy interlayer is effective to direct Li^0^ electrodeposition by virtue of favorable charge transfer or Li diffusivity across the interlayer and therefore suppress dendrite in LE systems, in accordance with previous findings.^[^ ^12^, ^13^ ^]^

**Figure 1:**
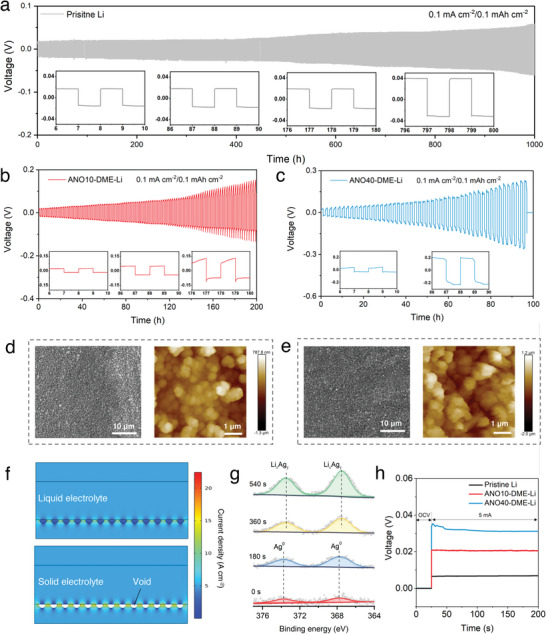
Evaluation of routine CRI‐alloy interlayer in SE system. Voltage profiles of symmetric cells employing a) pristine Li, b) ANO10‐DME‐Li, and c) ANO40‐DME‐Li under the current density of 0.1 mA cm^−2^ with a fixed plating/stripping duration of 1 h. SEM images and AFM topography images of d) ANO10‐DME‐Li and e) ANO40‐DME‐Li anodes. f) COMSOL simulations showing local current density concentrations in the vicinity of the edges of surface voids in LE and SE systems. g) High‐resolution XPS depth spectra of Ag 3d of the ANO10‐DME‐Li anode. h) Measurements of d.c. conductivity of pristine Li, ANO10‐DME‐Li, and ANO40‐DME‐Li anodes using blocking electrodes (more details for calculating electronic conductivities of the interlayers are described in the Experimental Section, Supporting Information).

Regarding the sulfide SE system, the implications of the as‐prepared CRI‐alloy interlayer for suppressing dendrites show paradoxical discrepancies compared with those in the LE system. The lithium argyrodite Li_6_PS_5_Cl SE was used in the ASSLBs due to its competent ionic conductivity (2.54 × 10^−3^ S cm^−1^) and facile synthesis procedure (Figure S8, Supporting Information). At a low current density of 0.1 mA cm^−2^, the Li|Li_6_PS_5_Cl|Li symmetric cell shows a stable voltage profile over 1000 h (**Figure** **1**a). Despite a slight increase in overpotential during cycling, no significant short‐circuit failure was observed. This is mainly attributed to the generation of a kinetical self‐limiting interface between Li_6_PS_5_Cl and Li^0^, which somewhat inhibits the continuous degradation of sulfide SEs.^[^ ^6^, ^16^ ^]^ As a comparison, the time‐synchronized voltage traces show considerable differences after the insertion of routine CRI‐alloy interlayers, where the hysteresis voltages of both ANO10‐DME‐Li and ANO40‐DME‐Li present a rapid increase during cycling (Figure 1b,c). As shown in Figure S9a, Supporting Information, the overpotentials of ANO10‐DME‐Li and ANO40‐DME‐Li increase to 44.4 and 174.3 mV after 80 h, respectively; in contrast, the pristine Li anode remains at a constant voltage polarization of ≈18 mV over 100 h. In addition, characteristic variations in the voltage plateau shape of CRI‐alloy‐protected Li anodes are enigmatic: the voltage plateau of ANO10‐DME‐Li holds almost flat and steady in the early cycles (e.g., 0–100 h) and then progressively transits into an exponential increase or decrease in the slope at the latter stages of cycling; in contrast, the voltage plateau of ANO40‐DME‐Li presents drastic polarization with “peaking” behavior (see Figure S9b, Supporting Information) throughout cycling. The general worsening traces of voltage profiles were also observed in other ANO*x*‐DME‐Li samples with higher concentration or longer immersion time, as displayed in Figure S10, Supporting Information.

Indeed, this peaking behavior of voltage polarization has been shown to correlate with inactive Li accumulation and the subsequent formation of pits, indicating non‐uniform Li^0^ deposition and dissolution during cycling.^[^ ^17^ ^]^ Revisiting the surface morphology of CRI‐alloy interlayers in Figure S4, Supporting Information, it can be determined that the polarization degree of voltage profiles is closely associated with the corresponding morphological roughness of CRI‐alloy interlayers. This phenomenon may account for the consensus that surface defect regions exhibit faster Li deposition kinetics and higher nucleation tendency. After the fast conversion‐alloy reaction, lots of artificial defects, including pits and protrusions, are generated on the surface of re‐engineered Li electrodes, which reduce the contact area between the SE separator and the Li anode, thereby increasing the local current density because of the tip effect of the electric field. As a result of such electrical constrictions, initial Li^0^ deposition preferably proceeds on these artificial defect sites, resulting in local nucleation and self‐amplifying behavior of dendrite growth.^[^ ^18^ ^]^ For example, although SEM images of ANO10‐DME‐Li and ANO40‐DME‐Li anodes indicate that they have similar morphology in terms of surface homogeneity and integrity, the topographic atomic force microscope (AFM) topography images map the average roughness of ANO10‐DME‐Li and ANO40‐DME‐Li anodes to 148 and 568 nm, respectively (see Figure 1d,e and Figure S11, Supporting Information). The ANO10‐DME‐Li surface contains smaller nanoparticles with more uniform spatial distribution and fewer cavities, while the higher concentration (40 mm) of ANO results in the formation of larger alloy particles that are prone to agglomerate into clusters. This slight difference in roughness between the two anodes is expected to affect the initial deposition morphology and thus the uniformity of subsequent repeated plating/stripping in the SE system. As evident by the time‐evolving morphological changes of these CRI‐alloy‐protected electrodes during cycling (see Figure S12, Supporting Information), the uniform Li deposition corresponds well to the flat voltage profile plateau, while rough surface means fluctuations of voltage polarization and uncontrolled Li deposition. Compared to the ANO40‐DME‐Li anode, the increase in short‐circuit time and relatively even Li deposition morphology of the ANO10‐DME‐Li anode implies that strict roughness control of re‐engineered Li electrodes is vital to the spatial homogeneity of electrochemical processes occurring on the Li electrode surface in the SE system.

These controlled experiments suggest that the Li deposition behavior related to the roughness of CRI‐alloy interlayers is different in LE and SE systems. Apparently, the LE system can tolerate an appropriate roughness of the re‐engineered Li electrode to achieve a uniform and dense Li deposition, whereas the SE system is more sensitive to defects or voids on the electrode surface, even with ultra‐small roughness of several hundred nanometers. To clarify this, we carried out COMSOL simulations to assess the impact of interfacial voids on local current densities in both LE and SE systems (Figure 1f and Figure S13, Supporting Information). In cells with LEs, the Li anode remains in contact with LEs despite the presence of voids on the Li foil surface, while discontinuities at the Li/SE interface are expected due to the absence of liquid wetting. As a result, at interfaces with poor or discontinuous electrical contact, electrochemical current detours around voids, resulting in local electrical constrictions near the edges of the defects/voids. Under an average cell current density of 0.5 mA cm^−2^, the local current density at void edges could be as high as 56.9 and 328.7 A cm^−2^ for LE and SE systems, respectively. Therefore, the resultant amplification effect of void/defect‐induced local current concentrations in the SE system is prone to render the formation of Li filaments via electromigration or an (electro)chemomechanical mechanism.^[^ ^19^ ^]^

Another anomaly that appears in the above voltage profiles is the increasing overpotential with repeated platting/stripping, consistent with the accumulation of interfacial resistance shown in Figure S14, Supporting Information. Considering that the shape of the voltage profile of ANO10‐DME‐Li remains steady over 0–100 h with limited dendrite accumulation (Figure S12, Supporting Information), it is reasonable to speculate that, in this case, persistent interfacial reactions at the Li/SE interface may be responsible for the ever‐increasing overpotential.^[^ ^20^ ^]^ This scenario resembles the interfacial evolution between Li_10_GeP_2_S_12_ and Li^0^, where the formation of mixed ionic‐electronic conductive (MIEC) interphases (i.e., Li*_x_* Ge alloy) leads to the continuous degradation of SEs and an increasing impedance.^[^ ^21^ ^]^ The composition distribution in the CRI‐alloy interlayer was further analyzed via X‐ray photoelectron spectroscopy (XPS) at different etching depths. For Ag 3d spectra shown in Figure 1g, peaks located at 367.7 and 373.7 eV are assigned to Ag 3d_5/2_ and 3d_3/2_ of metallic Ag, respectively, in the upper regions of the interlayer.^[^ ^22^ ^]^ With increasing sputtering to 360 s, the peaks shift downward to 367.5 and 373.5 V, indexed by the featured peaks of Li*_x_* Ag*_y_*. The peak intensities increasing with sputtering thickness indicate the vertically gradient distribution of Ag‐based species in the interlayer. Combined with the Li 1s spectra (Figure S15, Supporting Information), it can be determined that the CRI‐alloy interlayer exhibits a mosaic structure consisting mainly of inorganics, including LiOH, Li_2_CO_3_, Li_2_O, and Li*_x_* Ag*_y_*. The presence of metallic Ag at the surface regions of the interlayer would act as effective Li nuclei triggering preferential Li^0^ deposition on or inside the CRI‐alloy interlayer.^[^ ^15^, ^23^ ^]^ In addition, the Ag‐based species distributed vertically through the surface layer are expected to provide channels for leakage current to decompose sulfide SEs and promote direct Li^0^ nucleation inside the SE separator. As corroborated by direct current‐voltage measurements of these Li anodes (see Figure 1h), the ANO10‐DME‐Li and ANO40‐DME‐Li exhibit high electronic conductivities of 5.8 × 10^−3^ and 3.7 × 10^−3^ S cm^−1^, respectively. As a result of the electron‐conducting nature of CRI‐alloy interlayers, charge transfer reactions related to SE reduction and Li deposition/dissolution preferentially take place right between the interlayer and the SE separator, consistent with the observed nonuniform Li^0^ plating atop the re‐engineered anode surface (Figure S12, Supporting Information). The freshly plated active Li^0^ is in direct contact with sulfide SEs, resulting in the continuous reduction of sulfide SEs and accumulation of inactive Li^0^, which deviates from the original purpose of the protective layer design.

### 2.2 Tailoring CRI‐Alloy Interlayer for SE System

**Figure 2:**
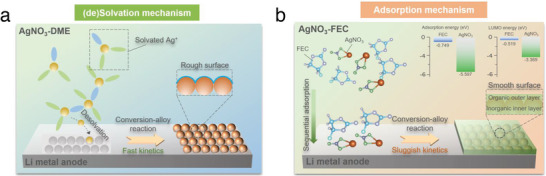
Schematic illustrations of the surface chemistry route to prepare the CRI‐alloy protected Li anodes based on a) (de)solvation mechanism and b) adsorption mechanism.

In light of the above systematic studies of the electrochemical processes and corresponding morphology evolution of CRI‐alloy‐protected Li anodes in LE and SE battery chemistries, it can be determined that an ideal CRI‐alloy interlayer for SE systems should avoid the formation of artificial hotpots and uncontrolled Li^0^ nucleation located at the SE/interlayer interface; in this regard, CRI‐alloy interlayers are proposed to be tailored to the SE system in terms of morphology as well as properties. Following this design principle, using FEC solvent instead of DME, we further adjusted the CRI‐alloy interlayer from the previous mosaic structure to a hierarchical gradient structure by regulating the conversion reaction kinetics from a (de)solvation model to an adsorption mechanism, yielding a smooth organic‐rich outer layer and a composition‐regulated inorganic‐rich inner layer composed mainly of lithiophilic Li*_x_* Ag*_y_* and electron‐insulating LiF (**Figure** **2**a,b). The tailored CRI‐alloy interlayer is expected to benefit from multiple merits, as follows:

- A smooth organic‐rich outer layer with negligible defects would avoid preferential Li deposition at artificial hotpots and maintain intimate ionic contact at the SE/Li interface.
- The Li*_x_* Ag*_y_* component with a high Li diffusion coefficient could decrease Li diffusion barriers within the CRI‐alloy interlayer and homogenize top‐down Li^+^ flux across the interface.
- The electron‐insulative LiF helps to build a potential gradient across the CRI‐alloy interlayer, which provides the driving force for Li^0^ deposition beneath the interlayer. In addition, LiF is known as a robust barrier to dendrite growth on the aspect of high surface energy.^8^, ^24^ ^]^

**Figure 3:**
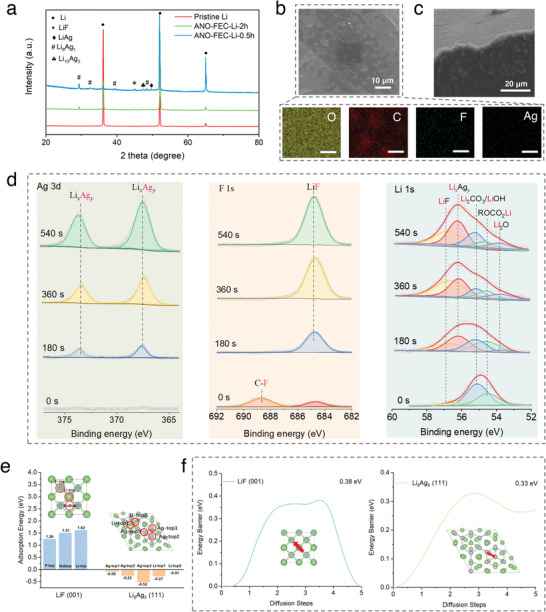
Tailoring CRI‐alloy interlayer for SE system. a) XRD patterns of pristine Li and ANO‐FEC‐Li anodes. b) Top‐view SEM image of ANO‐FEC‐Li anode (5 mm) and the corresponding EDS maps of O, C, F, and Ag (scale bars are 20 µm). c) Cross‐sectional SEM image of ANO‐FEC‐Li anode (5 mm). d) Ag 3d, F 1s, and Li 1s XPS depth spectra in the ANO‐FEC‐Li surface. e) Schematics of selected adsorption sites for investigations and the calculated adsorption energy of Li^+^ on the LiF(001) and Li_8_Ag_5_(111) surfaces. The insets are the top‐view of the adsorption sites toward Li^+^ on the 2 × 2 LiF(001) and 1 × 2 Li_8_Ag_5_(111) surfaces. f) The energy barriers of Li diffusion in the interfacial bulk phases of LiF(001) (left) and Li_8_Ag_5_(111) (right).

From the perspective of corrosion science, the possible rate‐determining step in the conversion reaction between AgNO_3_ and Li^0^ is the (de)solvation process of Ag^+^, which is dictated by solvent‐ion interactions.^[^ ^25^ ^]^ Although AgNO_3_ is difficult to dissolve in most organic aprotic solvents due to the large binding energy between NO_3_^−^ and Ag^+^, two electronegative oxygen atoms in a DME molecule can simultaneously coordinate with an Ag^+^, prompting the sufficient dissociation of AgNO_3_ followed by the formation of solvated Ag^+^. Simultaneously, the small dielectric constant (5.5) of DME allows DME molecules to interact weakly with Ag^+^ in the solvation sheath, which results in the formation of contact ion pairs in the ANO‐DME solution by coordinated NO_3_^−^ and promotes Ag^+^ desolvation.^[^ ^26^ ^]^ As a result, a rapid conversion reaction kinetics between solvated Ag^+^ and Li^0^ is established in the ANO‐DME case (as signaled by a few seconds of electrode discoloration in Figure S2, Supporting Information), triggering rapid nucleation of Li*_x_* Ag*_y_* particles, accompanied by drastic phase change that manifests as a defect‐rich and rough surface morphology (Figure 2a). In this regard, diluting the degree of Ag^+^ solvation is proposed to restrict the conversion‐alloy reaction kinetics between AgNO_3_ and Li^0^. Compared to DME with a high donor number (DN) (20 kcal mol^−1^), FEC with a low DN (7.9 kcal mol^−1^) shows inferior solubility for AgNO_3_.^[^ ^27^ ^]^ As identified by the inductively coupled plasma emission spectroscopy (ICP; Figure S16, Supporting Information), AgNO_3_ dissolved in FEC (referred to as ANO‐FEC) reaches saturation before 10 mm. According to the equilibrium AgNO_3_* ↔ Ag^+^_(sol)_ + NO_3_^−^_(sol)_ + ion pairs + higher aggregates (* indicates Li surface‐adsorbed AgNO_3_), using FEC instead of DME results in weak solvation of Ag^+^ and the equilibrium lies to the left.^[^ ^28^ ^]^ Thereby, the surface‐absorbed AgNO_3_ dominates the conversion reaction kinetics between AgNO_3_ and Li^0^ in the ANO‐FEC case. Moreover, due to the strong electron‐absorbing effect of the F functional group, FEC possesses a large dielectric constant (78.4), which hinders the subsequent desolvation of Ag^+^ as a result of strong solvent‐cation interactions.^[^ ^26^, ^29^ ^]^ Eventually, as illustrated in Figure 2b, the conversion‐alloy reaction kinetics is effectively constrained, as evidenced by a longer immersion decolorization time (Figure S17, Supporting Information), which provides an indispensable time condition for the sequential reduction of the active layer formers based on the adsorption model. Importantly, AgNO_3_ and FEC have distinct adsorption energies (−5.60 and −0.75 eV, respectively) and different lowest unoccupied molecular orbital (LUMO) energy levels (−3.37 and −0.52 eV, respectively), triggering their sequential adsorption and reduction to form an inorganic inner layer enriched with Li‐Ag and LiF (Figure S18, Supporting Information). As verified by XRD results (**Figure** **3**a), Li_8_Ag_5_ (mp‐1211140) is the main Li‐rich solid‐solution alloy interphase, similar to that presented on the ANO‐DME‐Li surface.^[^ ^30^ ^]^ The diffraction peak at ≈44.9° is ascribed to crystalline LiF (JCPDS No. 04–0857).

The surface morphologies of the ANO‐FEC‐Li electrodes with different soaking times were observed by SEM (Figure S19a, Supporting Information). As expected, due to sluggish kinetics in the ANO‐FEC solution, seconds of soaking time (e.g., 60 s) are insufficient to initiate the conversion reaction based on the adsorption model, as reflected by the fact that the treated Li anode maintains the same featureless morphology as the pristine Li anode. The electrode surface was covered with rough nanoparticles after soaking for 0.5 h, indicating a prior reduction of AgNO_3_ than the FEC molecule. Interestingly, as the soaking time was further increased to more than 2 h, the rough surface with nano‐granular structure converted into a smooth and continuous film‐like state, resembling the morphology of the Li anode pretreated with neat FEC solvent (Figure S19b, Supporting Information). Similar to the regulation of Li^+^ solvation sheath by FEC as an additive in LE systems, FEC undergoes decomposition reactions with Li^0^ because the weak C—F bonds in FEC molecules are easily broken by the electrostatic attraction of Li‐F, forming LiF and organic species (e.g., ROCO_2_Li and ROLi).^[^ ^31^ ^]^ Since organics have a relatively higher solubility in FEC solvents than inorganics, the preferential dissolution of organic species into the solvent and subsequent solvent evaporation result in the formation of the organic–inorganic hierarchical gradient structure. The energy‐dispersive spectroscopy (EDS) mapping of the ANO‐FEC‐Li anode confirms the uniform distribution of C, O, and F (Figure 3b). Significantly, the detectable Ag signal is negligible compared to the abundant Ag distribution on the ANO‐DME‐Li surface (Figure 20, Supporting Information), implying that Ag‐based species are mainly located beneath the organic components. The soaking time of 2 h was chosen as the optimized condition since the longer soaking time triggers organic species to continue to polymerize into clusters with disordered structures that would block Li^+^ transport.^[^ ^31b^ ^]^ The thickness of the tailored CRI‐alloy interlayer is estimated to be 4 µm, as shown in Figure 3c.

XPS depth profiles were collected to determine the composition distribution within the tailored CRI‐alloy layer governed by the adsorption‐conversion mechanism. Figure S21a, Supporting Information, displays the atomic concentration distribution along with the depth of the CRI‐alloy interlayer. The C content decreases sharply within the first 30 nm depth, then followed by a gradual decrease, corresponding to the vertical decrease of the organic content within the interlayer. Based on the detailed analysis of C 1s and Li 1s spectra (Figure 3d and Figure S21b, Supporting Information), the surface of ANO‐FEC‐Li mainly consists of organic moieties of C—C/C—H, C—F, and ROCO_2_Li. In addition, both Ag 3d and F 1s spectra corroborate the depth distribution of Li*_x_* Ag*_y_* and LiF (Figure 3d). Importantly, we can differentiate evident changes in the distribution of surface Ag species between ANO‐DME‐Li and ANO‐FEC‐Li; the absence of featured Ag peaks on the ANO‐FEC‐Li surface indicates that the thorough self‐alloying process is correlated to conversion kinetics, which is expected to impede the electronic conduction and protect the sulfide SE from electron attack. Consequently, the identified chemistries in the inorganic layer also include Li_2_O, Li_2_CO_3_, and LiOH, and in the inner region, Li‐Ag and LiF are the major components (Figure S21c, Supporting Information).

The benefits of combining Li*_x_* Ag*_y_* and LiF for composition regulation in the CRI‐alloy interlayer were clarified by density functional theory (DFT) calculations. The lattice planes of Li_8_Ag_5_ (111) and LiF (001) were selected as the target surface models to investigate the Li adsorption and migration kinetics on the surface because they exhibit the lowest surface energies (see Figure S22, Supporting Information). Li_8_Ag_5_ demonstrates lower adsorption energies than the F‐top site of LiF, indicating a higher affinity for Li^+^ (see Figure 3e). In addition, the climbing‐image nudged elastic band (CI‐NEB) method was employed to investigate the Li diffusion on the most stable adsorption sites. The corresponding energy profiles in Figure 3f reveals that the Li_8_Ag_5_ (111) surface exhibits a lower Li diffusion barrier (0.33 eV) than the LiF(001) surfaces (0.38 eV).^[^ ^32^ ^]^ Therefore, it can be concluded that Li_8_Ag_5_, with both the strong adsorption and low diffusion barrier toward Li, can concentrate Li^+^ on its surface and then boost rapid Li chemical diffusion across the CRI‐alloy interlayer, which overcomes the Li^0^ self‐diffusion limitation at the SE/Li interface.

As a further step, the interfacial supercell models with incoherent sharp interfaces based on the interfacial misfit minimization were set up to theoretically evaluate the effect of Li_8_Ag_5_ and LiF on dendrite suppression. Then interfacial properties, including the formation energy (E_f_), strain energy (*ζ*), interfacial energy (*σ*), and the work of adhesion (*W*_adh_) of the interlayer, were calculated for mechanical stability assessment (Figure S23, Supporting Information).^[^ ^33^ ^]^ The calculated results show that LiF can serve as a more rigid component of interphases than Li_8_Ag_5_ to suppress dendrite penetration due to its higher *σ* and E_f_ values. However, Li_8_Ag_5_, with a higher *W*_adh_ value than LiF, exhibits better wettability to the Li electrode, thus preventing contact loss at the Li/SE interface during cycling. These results show that the unique combination of the Li_8_Ag_5_ and LiF in the protective layer can complement each other in terms of wettability and dendrite suppression ability, which is expected to integrate enhanced Li^+^ adsorption, rapid Li chemical diffusion and high dendrite growth tolerance simultaneously to stabilize the Li anode.

### 2.3 Stable Lithium Plating and Stripping in SE System

**Figure 4:**
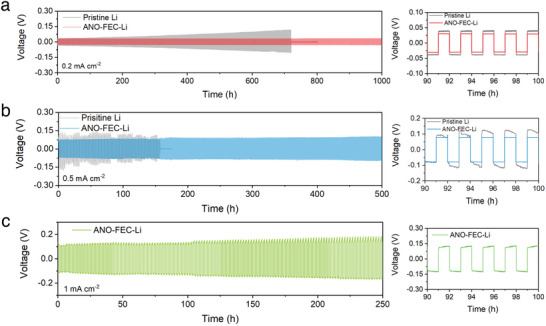
Stable lithium plating/stripping regulated by the tailored CRI‐alloy interlayer in the SE system. Voltage profiles as a function of time of symmetric cells with pristine Li and ANO‐FEC‐Li anodes under different current densities of a) 0.2, b) 0.5, and c) 1 mA cm^−2^ with a fixed plating/stripping duration of 1 h.

The implications of the tailored CRI‐alloy interlayer for the stability of Li plating/stripping in the SE system were probed using Li‐Li symmetric cells. **Figure** **4**a–c shows the galvanostatic time‐synchronized voltage profiles at different current densities with a fixed plating/stripping duration of 1 h. The Li|Li_6_PS_5_Cl|Li cells exhibit increasing overpotentials after the initial voltage hysteresis of 32.8 mV, and sudden failure occurs after 719 h of cycling at 0.2 mA cm^−2^/0.2 mAh cm^−2^. With the current density and areal capacity increase to 0.5 mA cm^−2^/0.5 mAh cm^−2^, significant voltage polarization and “soft” short circuits were observed from the voltage traces of symmetric cells employing the pristine Li anode, and eventual “hard” short circuit occurs after 157 h, indicating unstable Li plating/stripping at a high current density. In contrast, incorporating the tailored CRI‐alloy interlayer enables prolonged cycling with steady and flat voltage profiles of symmetric cells for over 1000 and 500 h at current densities of 0.2 and 0.5 mA cm^−2^, respectively. In addition, the smaller and flatter average overpotentials of ANO‐FEC‐Li throughout cycling compared to that of the pristine Li anode imply faster Li^+^ transport kinetics across the CRI‐alloy interlayer, consistent with the results of EIS experiments where the ANO‐FEC‐Li shows smaller charge transfer resistance before and after cycling compared to its counterpart (Figure S24, Supporting Information). Even at a current density of 1 mA cm^−2^, the ANO‐FEC‐Li symmetric cells still maintain excellent compatibility against Li_6_PS_5_Cl, and the overpotentials remain steady (as low as 137.2 mV) for over 250 h, whereas pristine Li cannot operate at this condition. Moreover, a harsher half‐cycle capacity of 2 mAh cm^−2^ at the current density of 0.5 mA cm^−2^ was performed for the ANO‐FEC‐Li symmetric cells (Figure S25, Supporting Information). We found that it possible to reversibly plate for a total capacity of over 364 mAh cm^−2^, corroborating the effective regulation in Li plating/stripping by the tailored CRI‐alloy interlayer. The availability of the tailored CRI‐alloy layer applied in other sulfide‐based SE systems was demonstrated in LGPS‐based symmetric cells, where the increasing overpotential caused by the MIEC interface is adjusted to a steady polarization voltage over 500 h (Figure S26, Supporting Information).

**Figure 5:**
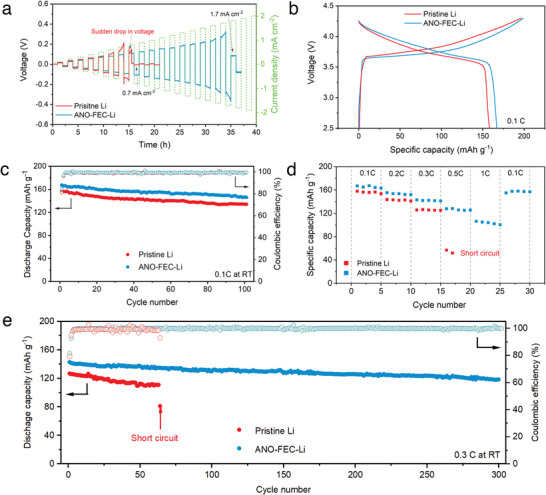
a) Galvanostatic cycling of symmetric cells with pristine Li and ANO‐FEC‐Li anodes with step‐increased current densities of 0.1 mA cm^−2^ with a constant half‐cycle duration of 1 h. b) Galvanostatic charge–discharge profiles of ASSLBs employing pristine Li and ANO‐FEC‐Li anodes at the current density of 0.1 C (RT). c) Cycling performances of ASSLBs at 0.1 C. d) Rate capability for ASSLBs with pristine Li and ANO‐FEC‐Li anodes. e) Cycling performances of ASSLBs for 300 cycles at 0.3 C (RT).

To further evaluate the improved dendrite tolerance regulated by the tailored CRI‐alloy interlayer in the SE system, dendritic distributions in SE separators were characterized by SEM after the above symmetric cells cycling at 0.5 mA cm^−2^ (Figure S27, Supporting Information). For the pristine Li case, abundant accumulations of dead/inactive Li and dendrites were observed at the SE/Li interface, especially in the surface defect regions. Moreover, distinct dendrite propagation through the entire SE separator can be captured from the cross‐sectional SEM of the SE separator, accompanied by mechanical spallation of the SE separator, posing a short circuit danger. As a comparison, the cycled SE separator paired with the ANO‐FEC‐Li anode exhibits dendrite‐free morphologies from the surface to the bulk, allowing uniform Li plating/stripping at a relatively high current density. In addition, CCD (defined as the maximum endurable current density of the cell without a short circuit) measurement is adopted with step‐increased current densities of 0.1 mA cm^−2^ with a constant half‐cycle duration of 1 h (see **Figure** **5**a). The pristine Li shows a low CCD of 0.7 mA cm^−2^ as evidenced by the sudden fluctuation of overpotential, consistent with the degraded cycling stability at high current densities in galvanostatic plating/stripping measurements. Contrast to this, the CCD in ANO‐FEC‐Li symmetric cells increases to 1.7 mA cm^−2^ with a high areal capacity of 1.7 mAh cm^−2^, corroborating that inserting the tailored CRI‐alloy interlayer increase dendrite tolerance in the SE system. It is noteworthy that the LiF‐protected anode (FEC‐Li) obtained from a pure FEC solution shows limited improvement in CCD of symmetric cells (Figure S28a, Supporting Information). Despite the FEC‐Li symmetric cells being able to cycle stably at a current density of 0.2 mA cm^−2^, they display higher plating/stripping overpotentials compared to those of ANO‐FEC‐Li and pristine Li. (Figure S28b, Supporting Information). This is primarily due to the low ionic conductivity and poor wettability of LiF, resulting in increased interfacial resistance. Consequently, as the current density increases to 0.5 mA cm^−2^, the plating/stripping overpotential continues to rise and ultimately becomes unstable, indicating the accumulation of lithium dendrites at the interface (Figure S28c,d, Supporting Information). In this regard, the Li*_x_* Ag*_y_* phase plays a crucial role in reducing interfacial resistance and promoting uniform Li deposition/stripping at high current densities.

To further validate the availability of the tailored CRI‐alloy‐protected anodes applied in practical full batteries, we probed the electrochemical performances of ASSLBs using Li_3_B_11_O_18_‐coated Li(Ni_0.6_Co_0.2_Mn_0.2_)O_2_ (NCM622) cathode (more details see Figure S29, Supporting Information) and pristine Li or ANO‐FEC‐Li anodes. As shown in Figure 5b, the ANO‐FEC‐Li|SE|NCM622 cell displays a higher initial discharge capacity of 167.4 mAh g^−1^ and Coulombic efficiency (CE) of 84.2% than that of the cell with the pristine Li anode (158.2 mAh g^−1^ and 80.6%, respectively) at the current density of 0.1 C. After 100 cycles, the cells show capacity retentions of 84.9% and 87.2% for pristine Li and ANO‐FEC‐Li, respectively (Figure 5c). The improved electrochemical performances of ASSLBs with the ANO‐FEC‐Li anode are ascribed to the enhanced Li^+^ transfer kinetics across the tailored CRI‐alloy interlayer during cycling, consistent with the EIS results shown in Figure S24, Supporting Information. Notably, the significant improvement in electrochemical performances by engineering the tailored CRI‐alloy interlayer is more pronounced in the aspect of rate capability (see Figure 5d). In the pristine Li case, the capacity drops significantly upon increasing the current density to 0.5 C, accompanied by a premature short circuit. By contrast, cells employing the ANO‐FEC‐Li anode exhibit good rate capability; with the current rate step‐increasing from 0.1 to 1 C, the capacity ranges from 164.4 to 106.5 mAh g^−1^, corresponding to a capacity retention of 64.8% with a 10‐fold increase in current. Furthermore, the cell with the ANO‐FEC‐Li anode presents excellent cycling stability with a capacity retention of 82.7% over 300 cycles at 0.3 C, while the pristine Li completely fails by a short circuit at 64 cycles, further corroborating the superiority of the tailored CRI‐alloy interlayer in terms of interface regulation at high current densities (Figure 5e).

### 2.4 Origin of Tailored CRI‐Alloy Layer for Dendrite Suppression

**Figure 6:**
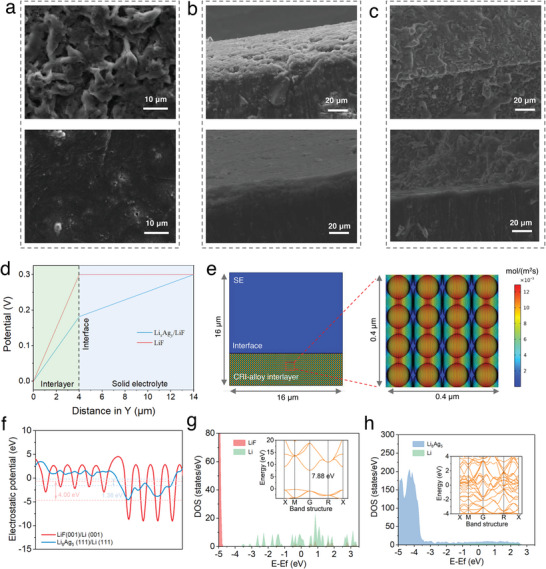
Origin of the tailored CRI‐alloy layer for dendrite suppression. a) Top‐view and b) cross‐sectional SEM images of pristine Li (top) and ANO‐FEC‐Li (down) after initial electrodeposition with 2 mAh cm^−2^ of Li^0^. c) Cross‐sectional SEM images of the pristine Li/Li_6_PS_5_Cl (top) and ANO‐FEC‐Li/Li_6_PS_5_Cl (down) interfaces after full ASSLBs cycling thoroughly at 0.3 C at RT. d) Potential distribution for the Li*_x_* A*_g_*/LiF and LiF interlayers under a pre‐set overpotential of 0.3 V. e) FES of the cross‐sectional distribution of Li^+^ flux along the Y direction in the Li*_x_* A*_g_*/LiF interlayer. f) Electrostatic potential profiles of Li_8_Ag_5_(111)/Li(111) and LiF(001)/Li(001) interfaces. Density of states (DOS) of g) LiF(001)/Li(001) and h) Li_8_Ag_5_(111)/Li(111) interfaces. The band structures of LiF and Li_8_Ag_5_ unit cells are shown in the insets of (g) and (h), respectively.

To elucidate the reasons for the observed improvement in Li plating/stripping stability and dendritic suppression capability by engineering the tailored CRI‐alloy interlayer in the SE system, we first conducted the morphological analysis after initial electrodeposition with 2 mAh cm^−2^ of Li^0^. As shown in **Figure** **6**a, the deficiency of active nucleation sites on pristine Li leads to chaotic and nonuniform Li^0^ deposition, whereas the plated ANO‐FEC‐Li surface shows a well‐preserved smooth morphology similar to that of the pristine state. The cross‐sectional view of plated Li anodes shows that Li^0^ is plated on the surface of the pristine Li anode, while in the ANO‐FEC‐Li case, the deposition occurs underneath the interlayer with a dense morphology (Figure 6b). The plated Li^0^ thickness (≈10 µm) is in accord with that expected for the quantity of Li deposited. It is noted that the layer‐neath deposition behavior of Li^0^ for the ANO‐FEC‐Li anode isolates the sulfide SE from active Li^0^ and hence protects sulfide SE from continuous degradation, significantly different from the unstable interface deposition in the ANO‐DME‐Li case. The low electronic conductivity (1.43 × 10^−4^ S cm^−1^) of the ANO‐FEC‐Li anode (Figure S30, Supporting Information) may be responsible for this unique deposition behavior because there are fewer ion/electron junctions in the tailored CRI‐alloy interlayer than there would be with an electron‐conducting interlayer. For the Li electrodissolution process (of which SEM images are shown in Figure S31 Supporting Information), the pristine Li anode presents a rough and wrinkled surface with many voids and grooves after initial stripping, ascribed to the limitation of Li^0^ self‐diffusion at the high current density, as well demonstrated in the previous research.^[^ ^10^ ^]^ The incorporation of Li*_x_* A*_y_* with a low Li diffusion barrier in the interlayer facilitates extending Li mass transport from the Li^0^ self‐diffusion to rapid chemical diffusion, thus maintaining the ANO‐FEC‐Li surface smooth and flat without voids. Significantly, these voids are expected to accumulate on repeated plating/stripping that would trigger local electrical constrictions and resultant dendrite growth. According to cross‐sectional SEM images of the Li/SE interface, after the full ASSLBs cycle completion at 0.3 C (see Figure 6c), the plated Li^0^ layer without an interlayer shows a porous honeycomb morphology, which would lead to Li pulverization and contact loss at the interface. By contrast, the plated Li^0^ in the ANO‐FEC‐Li case is dense and smooth, ensuring interfacial integrity even after 300 cycles.

To better elaborate the homogeneous Li^0^ deposition behavior beneath the tailored CRI‐alloy interlayer, the finite element simulation (FES) was performed to visualize the distribution of the electric potential field and the Li^+^ flux across the Li/SE interface. As shown in Figure S32, Supporting Information, incorporating LiF into the Li*_x_* Ag*_y_* alloy interlayer results in a more uniform electric field in the entire region compared to the common LiF interlayer. Based on the cross‐sectional potential profile of the protective layer along the normal Y direction (see Figure 6d), an electrical potential gradient is established inside the tailored CRI‐alloy layer, and the combination of Li*_x_* Ag*_y_* and LiF contributes to a gentler voltage drop than the sole LiF component, which facilitates the decrease in overpotentials during cycling.^[^ ^34^ ^]^ In addition, the distribution of the Li^+^ flux in the tailored CRI‐alloy interlayer was simulated in Figure 6e. Due to the significant difference in Li diffusion coefficients between LiF and Li*_x_* Ag*_y_*, the Li^+^ flux in the tailored CRI‐alloy interlayer can be redistributed and predominantly distributed on the Li*_x_* Ag*_y_* phase instead of LiF, allowing fast and uniform Li diffusion across the interlayer.^[^ ^35^ ^]^ As a result, via regulating CRI‐alloy layer components, the uniformly distributed electrical potential field and Li^+^ flux are established at the interface, thereby allowing dendrite‐free Li deposition.

Furthermore, the electric properties across the interface were reflected by the planar‐average electrostatic potential (ESP) profiles (see Figure 6f). Electrons in the Li^0^ layer on interface exhibit lower work function compared to those in the sole LiF or Li_8_Ag_5_ layers, implying electrons are prone to transfer from the bulk Li anode to the SE separator, accompanied by the formation of a build‐up electric field in the vertical direction of the interface.^[^ ^33^ ^]^ The strength of the internal electric field can be evaluated by the difference between the macro‐average ESP that approximates the Fermi level of the component on each side of the interface (represented by the dashed lines in Figure 6f). LiF(001)/Li(001) exhibits a wider macro‐average ESP gap of 4.00 eV than 1.36 eV in Li_8_Ag_5_(111)/Li(111), indicating a higher energy barrier for electron flow tunneling across the LiF/Li interface. The energy band diagrams shown in the insets in Figure 6g also confirm that LiF is identified as an insulator with a wider energy gap 7.88 eV, while Li*_x_* Ag*_y_* is a good electronic conductor without band gap. Moreover, the density of states (DOS) of Li_8_Ag_5_ is comparable to the feature of the Li metal of the interface around the Fermi level, implying that Li_8_Ag_5_ cannot effectively block electron injection from the bulk Li to the SE separator (Figure 6h). Hence, mobile electrons are likely to combine with Li^+^ to deposit in the inner or top region of Li_8_Ag_5_. However, the relatively weak DOS characteristic of LiF than that of the Li part of the interface is expected to inhibit electron tunneling across the interlayer and thus render the Li deposition beneath the protective layer (see Figure 6g), consistent with the ESP analysis. Of course, it cannot be completely ruled out that Li^+^ is initial reduced on the surface of ANO*x*‐FEC‐Li, and then inward‐deposited beneath the layer driven by the reversible solid‐solution‐based alloy phase change.^[^ ^14b^ ^]^ However, this hypothesis fails to explain our experimental observations in the ANO*x*‐DME‐Li case.

**Figure 7:**
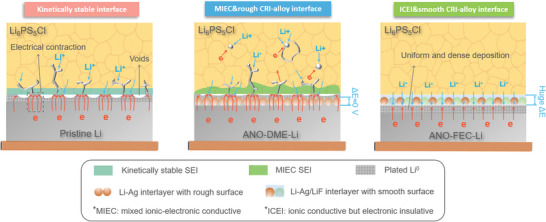
Schematics of Li deposition and dendrite growth scenarios at various interfaces, including kinetically sable interface, MIEC&rough CRI‐alloy interface, and ICEI&smooth CRI‐alloy interface.

To sum up, the reasonable Li deposition and dendrite growth scenarios at various interfaces discussed above are established, as shown in **Figure** **7**. In terms of the pristine Li case, the generated kinetics‐limiting interphase with Li_6_PS_5_Cl enables stable Li plating/stripping at a low current density (e.g., 0.1 mA cm^−2^). However, at high current densities, when the Li migration rate exceeds the Li replenishment rate by Li^0^ diffusion or deformation at the SE/Li interface, Li vacancies and resultant voids form on the electrode surface and accumulate during cycling, ultimately resulting in dendrite formation on plating. In addition, according to classical homogeneous nucleation theory, dendrite growth is related to the ratio of diffusion coefficient *D* to deposition flux *F* (*D*/*F*).^[^ ^36^ ^]^ Upon the deposition rate (i.e., current density) being faster than the diffusion rate, inhomogeneous nucleation occurs. In both mechanisms, the Li diffusion rate is the crucial determining factor for dendrite growth. Inserting a CRI‐alloy interlayer at the Li/SE interface is expected to eliminate the surface diffusion barrier and extend Li replenishing from vacancy diffusion to chemical diffusion, thus obviating void accumulation and inhomogeneous nucleation at a high current density. However, the rapid kinetics of common conversion reactions based on the (de)solvation mechanism triggers pit corrosion with many artificial defects appearing on the surface of the CRI‐alloy interlayer. The resultant local current contraction caused by these artificial defects will be amplified in the SE system compared to that in the moistening LE system, resulting in preferential dendrite nucleation and propagation at artificial hotpots. In addition, the electronic conductive alloy interphases cause electron injection from bulk Li to the SE separator, which disrupts the established dynamical equilibrium at the Li/Li_6_PS_5_Cl interface and makes the transition to an MIEC interface where the local potential to below 0 V versus Li^+^/Li.^[^ ^37^ ^]^ As a result, the continuous electronic degradation of sulfide SEs, accompanied by dendrite nucleation on/inside the CRI‐alloy interlayer or inside the SE separator, causes a premature short circuit of the symmetric cell. In this case, the diffusion barrier theory is invalid because dendrite growth on the interlayer is a heteroepitaxial growth process. Therefore, the MIEC alloy interlayer acts as a substrate for Li^0^ deposition, or more precisely, for prominent dendrite growth, instead of a protective shield. In contrast, by regulating the conversion reaction kinetics from the (de)solvation mechanism to an adsorption model, both the morphology and composition of the CRI‐alloy interlayer are adjusted to form an organic–inorganic hierarchical gradient structure. The organic‐rich outer layer ensures a smooth interlayer surface, which prevents preferential dendrite deposition and growth at the artificial defects. The functionality of inorganic components is role‐assigned but creates a synergistic effect. On the one hand, Li*_x_* Ag*_y_* is identified as a lithiophilic phase with enhanced Li^+^ adsorption and Li diffusivity that homogenize Li^+^ flux across the interlayer. On the other hand, the embedded LiF builds an indispensable potential gradient across the CRI‐alloy interlayer, promoting Li migration through the interlayer. As a result, the synergy of Li*_x_* Ag*_y_* with a low Li diffusion barrier and LiF with electron‐blocking property enables dendrite‐free and dense lithium deposition beneath the CRI‐alloy interlayer that protects sulfide SEs from the as‐mentioned electronic degradation. It should be emphasized that although LiF can be integrated into the interlayer using metal fluorides (e.g., AgF and ZnF_2_), the common synthetic method of CRI‐alloy interlayers applied in LE systems may not be directly replicated in the SE system because the surface roughness is uncontrollable based on the (de)solvation mechanism. In this regard, a sequential adsorption‐conversion model that controls the kinetics of the conversion reaction is of importance to the integration of smooth surface morphology, low diffusion barrier, and appropriate gradient potential in the CRI‐alloy layer specialized for ASSLBs.

## 3 Conclusion

In summary, the correlations between the properties of prevalent CRI‐alloy interlayers and dendrite growth in ASSLBs are established. We convincingly demonstrate that dendrite tolerance in ASSLBs is susceptible to the surface roughness and electronic conductivity of the CRI‐alloy interlayer. The typical surface chemistry route based on the (de)solvation mechanism to prepare the CRI‐alloy interlayer in LE chemistries may be inapplicable to the sulfide‐based SE system because pit corrosion kinetics is challenging to control. This puzzle is reasonably solved by tailoring the roughness and composition of the CRI‐alloy layer based on an adsorption‐conversion model, yielding a hierarchical gradient structure with a void‐free organic‐rich outer layer and a composition‐regulated inorganic‐rich inner layer composed mainly of lithiophilic Li*_x_* Ag*_y_* and electron‐insulating LiF. The local electrical constrictions and resultant dendrite growth caused by artificial defects are effectively inhibited via smoothening interlayer surface. Meanwhile, the excellent Li^+^ affinity and Li diffusivity of Li*_x_* Ag*_y_* overcome Li^0^ self‐diffusion limitation at the interface, while the LiF component with the electron‐blocking property confines electron tunneling into the SE separator. Taken together, the tailored CRI‐alloy interlayer integrates favorable roughness, conductivity, and diffusion properties simultaneously, enabling dendrite‐free Li deposition beneath the interlayer. Superior electrochemical performances were demonstrated with a high CCD of 1.7 mA cm^−2^ in symmetric cells and prolonged electrochemical cycling (300 cycles with a capacity retention of 82.7%) in the full ASSLBs. Given the existence of various available Li‐alloyable metal compounds and taking account of their rich surface science chemistry, the exploration of interactions with solvents based on the adsorption mechanism deserves further advance.

## Conflict of Interest

The authors declare no conflict of interest.

## Supporting information

Supporting Information

## Data Availability

The data that support the findings of this study are available from the corresponding author upon reasonable request.
