# Supplementary material for: Tailoring Conversion‐Reaction‐Induced Alloy Interlayer for Dendrite‐Free Sulfide‐Based All‐Solid‐State Lithium‐Metal Battery
Source: Adv Sci (Weinh). 2023 Apr 21;10(19):2300985. doi: 10.1002/advs.202300985 (PMC10323657; doi:10.1002/advs.202300985)
Supplement: Supplementary file 1 — Supporting Information [file ADVS-10-2300985-s001.pdf]

ADVANCED SCIENCE
Open Access

Supporting Information

for *Adv. Sci.*, DOI 10.1002/advs.202300985

Tailoring Conversion-Reaction-Induced Alloy Interlayer for Dendrite-Free Sulfide-Based All-Solid-State Lithium-Metal Battery

*Yuhao Liang, Chen Shen, Hong Liu, Chao Wang, Dabing Li, Xiaoxue Zhao and Li-Zhen Fan**

Supporting Information

Tailoring Conversion-Reaction-Induced Alloy Interlayer for Dendrite-Free Sulfide-Based All-Solid-State Lithium-Metal Battery

*Yuhao Liang, Chen Shen, Hong Liu, Chao Wang, Dabing Li, Xiaoxue Zhao and Li-Zhen Fan**

Y. H. Liang, H, Liu, C. Wang, H, Liu, D. B. Li, X. X. Zhao, Prof. L.-Z. Fan
Beijing Advanced Innovation Center for Materials Genome Engineering
Beijing Key Laboratory for Advanced Energy Materials and Technologies
University of Science and Technology Beijing
Beijing, 100083, China
E-mail: fanlizhen@ustb.edu.cn

C. Shen
Institute of Materials Science
Technical University of Darmstadt
Darmstadt, 64287, Germany

Methods

Preparation of CRI-alloy-protected anodes:

Lithium metal foil (99.9%, China Energy Lithium Co., Ltd.; 500 μm thickness) was first polished to remove surface contaminants using a nylon brush in the aprotic solvent of tetrahydrofuran and then dried before use. $AgNO_3$ was purchased from Alfa Aesar and mechanically ball-milled at 100 rpm for five hours. Then, different concentrations (5,10, 40, and 100 mM) of $AgNO_3$ were dispersed in dimethyl ether (DME) solvent and stirred vigorously for several hours until the solution became clear. The ANO-DME-Li anodes were fabricated by soaking the Li foil in the prepared solution for different times (optimized at 20 s) to induce the conversion reaction. Upon removal from the $AgNO_3$-DME solution, the residual impurities on the treated Li foil were carefully rinsed three times with DME. After drying at 60 ^oC overnight, routine CRI-alloy-protected anodes (ANO-DME-Li) were obtained and cut into 16 mm circles for further investigation. As for the preparation of the tailored CRI-alloy-protected anode (ANO-FEC-Li), using $AgNO_3$ dissolved in fluoroethylene carbonate (FEC) instead of $AgNO_3$-DME serve as the reaction solution. The concentration of $AgNO_3$ was determined to be 5 mM because of its ultra-low solubility in FEC. Drying at room temperature to prevent the formation of rough surfaces. The LiF-protected anode (FEC-Li) is also obtained by soaking Li foil in a pure FEC solution for 2 h as a contrast sample. All preparation procedures were carried out in an argon-filled glove box with < 1ppm oxygen and moisture.

Preparation of Li_6PS_5Cl SE:

Li_6PS_5Cl was synthesized via mechanical milling technique and subsequent heat-treatment. Li_2S (Alfa, 99.98%), P_2S_5 (Macklin, 99%), and LiCl (Sigma Aldrich, 99.99%) were mixed thoroughly with an appropriate stoichiometric ratio and then

mixed by the ball-milling procedure in a jar mill apparatus at a rotation speed of 510 rpm for 10 h. Subsequently, the obtained mixture was pressed into pellets by cold pressing and then sealed into quartz tubes for the following annealing treatment at 550 °C for 5 h. Finally, the ceramic pellets were ground into powder to obtain the ultima Li_6PS_5Cl SE. All procedures are carried out under an argon-protective atmosphere.

Characterizations:

XRD measurements were carried out on a Rigaku D/max-RB powder X-ray diffractometer with Cu Kαradiation in a 2θ range of 10−90°. The morphologies and mappings were obtained using the SEM technique (FE-SEM, JSM 6330). The roughness properties of the electrode surface were investigated on an AFM (Bruker ICON). The XPS samples were sealed in a vial before being quickly transferred to the chamber of an XPS Microprobe system for analysis (AXIS Ultra DLD, Kratos). The solubility of $AgNO_3$ in solvents was determined by inductively coupled plasma atomic emission spectrometry (ICP-OES, Perkin-Elmer ICP Optima 2000DV).

Electrochemical measurement:

Impedance and lithium plating/stripping studies were performed in the Li-Li symmetric cells using liquid electrolyte (LE) or Li_6PS_5Cl solid electrolyte (SE). The LE symmetric cells were assembled as a 2032 coin-type with 40 µl LE [1 M lithium bis(trifluoromethane)sulfonimide (LiTFSI) in a mixture of dioxolane/dimethoxyethane (DOL/DME) (1:1 vol)]. Celgard 2400 polypropylene (25 mm) was used as the separator in the coin cells. The Li electrodes were cut into 10 mm diameter disks placed on each side of the separator. Solid-state Li-Li symmetric cells were fabricated by a custom-made die mold composed of Ti rods and a polyether ether ketone (PEEK). Firstly, the SE layer was prepared by cold pressing preformed

Li_6PS_5Cl powders (120 mg) at 2 tons (~250 MPa) to mold it into a pellet (10 mm diameter) at 25 °C in an Ar-filled glovebox. Then, the SE separator was sandwiched with two pristine or treated Li disks (7 mm diameter) under the assembled pressure of 25 MPa. The assembly of ASSLBs is similar to that of solid-state symmetric cells. Composite cathode (10 mg, coresponding to mass loading of 13.93 mg cm^{-2}) composed of $Li_3B_{11}O_{18}$-coated $LiNi_{0.6}Co_{0.2}Mn_{0.6}O_2$ materials, Li_6PS_5Cl powders and vapor-grown-carbon-fibers (VGCF) at a weight ratio of 70:30:1 was spread on one side of the SE pellet, followed by pressing at 3 tons (~380 MPa). A piece of pristine Li or protected Li foil (7 mm diameter, 25 MPa) was put onto the other side of the SE pellet. ASSLBs were cycled between 2.5-4.3 V (versus Li^+/Li) under a working pressure of 5 MPa. The electrochemical impedance spectroscopy (EIS) technique was conducted using a CHI660B electrochemical workstation at a 10 mV amplitude over the frequency range of 0.1-10^5 Hz. The electronic resistivity of the protection layers was measured by examining the voltage response to a direct current (5 mA) on the cells with the pristine or treated Li foils sandwiched between two stainless steel blocking electrodes. The calculation formula is:

$$\rho = \frac{R \cdot S}{L} = \frac{U \cdot S}{I \cdot L}$$

Where L is the thickness of the protection film; I is applied current; S is area of the contact between stainless steel and the protecition film; U is average voltage increse.

Computational methods:

The spin polarized first-principles calculations based on the density function theory (DFT) were performed to investigate the molecular [i.e., highest occupied molecular orbital (HOMO) and lowest unoccupied molecular orbital (LUMO)] and the adsorption energies.[1, 2] For the calculations of molecular orbitals, Beck's three-parameter hybrid functional combined with Lee-Yang-Parr correlation (B3LYP)

functional was used for the exchange-correlation energy. Afterward, VMD and Multiwfn, two open-source software programs, were used for the graphical processing and rendering of the obtained molecular orbital maps. For the calculations of adsportion energies, the generalized gradient approximation (GGA) with the Perdew-Burke-Ernzerhof (PBE) functional was used to describe the exchangecorrelation energy implemented in the the Vienna Ab initio Simulation Package (VASP).[3] Projector Augmented Wave (PAW) methods were employed for the pseudopotentials. The energy cutoff for the plane wave basis was set as 450 eV and the convergence criterion of geometry relaxation was 0.02 eV $Å^{-1}$ in force. The Brillouin zones (BZ) were sampled by K point grids of 2π × 0.02 $Å^{-1}$. A vaccum of 15 Å was added along the direction normal to the atomic slab. Transition states of Li^+ diffusion and kinetics barriers were determined by the CI-NEB method implemented in the VASP code. The ΔG value can be determined as follows: ΔG = ΔE + ΔZPE −TΔS, where ΔE is the adsorption energy, ΔZPE is the change in zero-point energies, T is the temperature (T =298.15 K), and ΔS is the change of entropy. The adsorption energy ΔE is defined as: $\Delta E = E^{*}_{ads.} - (E^{*} + E_{ads.})$, where *ads. and * denote the adsorption of adsorbate on the substrates and bare substrates, $E_{ads.}$ denotes the energy of adsorbate. Li_8Ag_5 and LiF are in a cubic phase with space $I\overline{4}3m$ and Fm-3m with the optimized lattice constants of 9.61 and 4.08 Å, respectively, which are very close to the experimental values.

Current density distribution in the vicinity of voids, Li^+ transport and electrostatic field simulations were implemented by the COMSOL multiphysics. The simulations were performed in a 2D geometry for a cell with 10 μm thick Li ectrodes sandwiching a 2 μm thick slab of SE. The external surface of one of the Li electrodes is connected to a current source and the external surface of the other Li electrode is connected to a current sink. Faraday's law and current density were coupled at the electrode interface. The ionic conductivities of LE, SE, LiF, and Li_xAg_y are 1.1 × 10^{-2} S cm^{-1}, 2.3 × 10^{-3} S cm^{-1}, 1 × 10^{-7} S cm^{-1}, and 6×10^{-2} S/cm^{-1}, respectively. The Li diffusion coefficients in the LE, SE, LiF, and Li_xAg_y are 2.93 × 10^{-6} cm^2/s, 1 × 10^{-9} m^2/s, 4 × 10^{-13} m^2/s, and 4 × 10^{-8} m^2/s, respectively. The plating voltage difference between the two ends of the model was set as 0.3 V.

Supplementary Figures

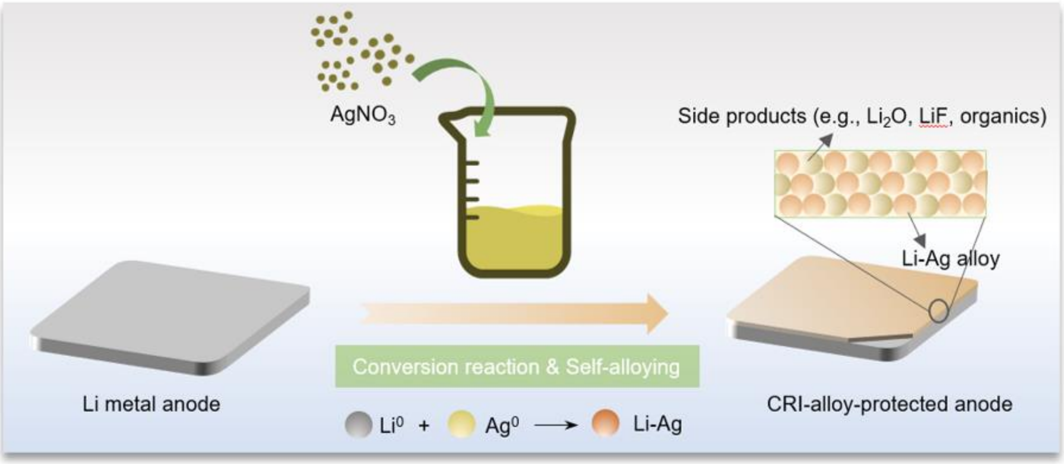

Figure S1. Schematic illustration of the facile solution-based surface chemistry route to prepare the CRI-alloy-protected Li anode.

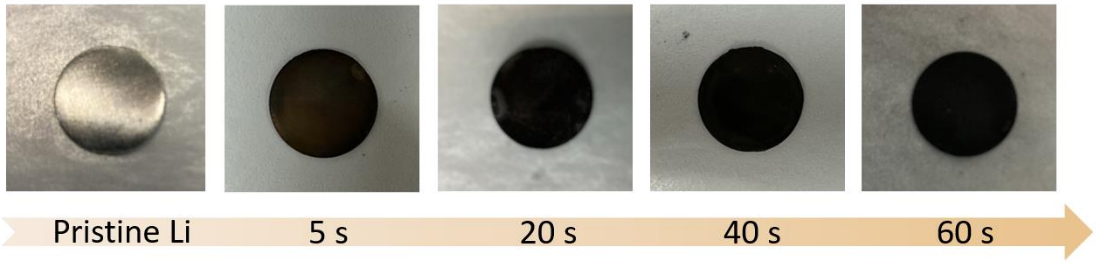

Figure S2. Optical photographs of Li anodes after immersion in ANO-DME solution for different times. The Li anode quickly changes color to brown after 5 s of immersion and maintains black after soaking more than 20 s.

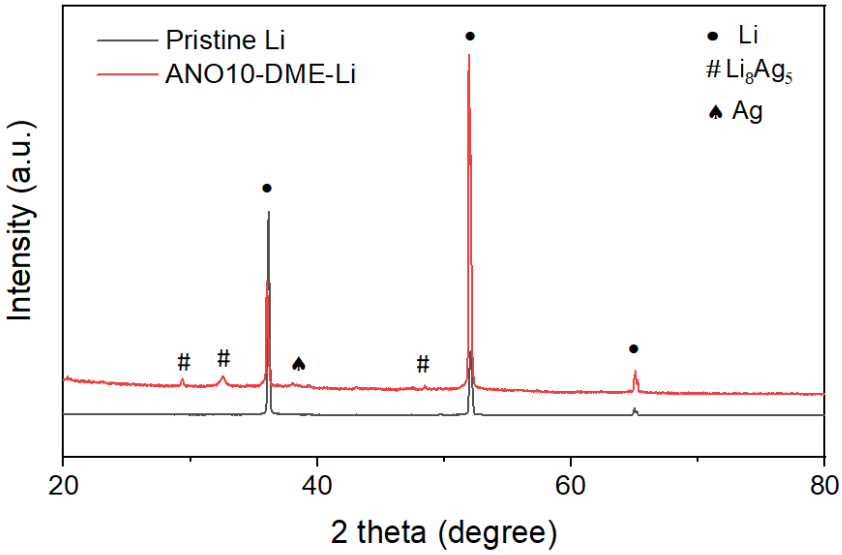

Figure S3. XRD spectra of the pristine Li and ANO10-DME-Li anodes. Li_8Ag_5 (mp-1211140) is identified as the main solid-solution alloy interphase on the ANO10-DME-Li surface.

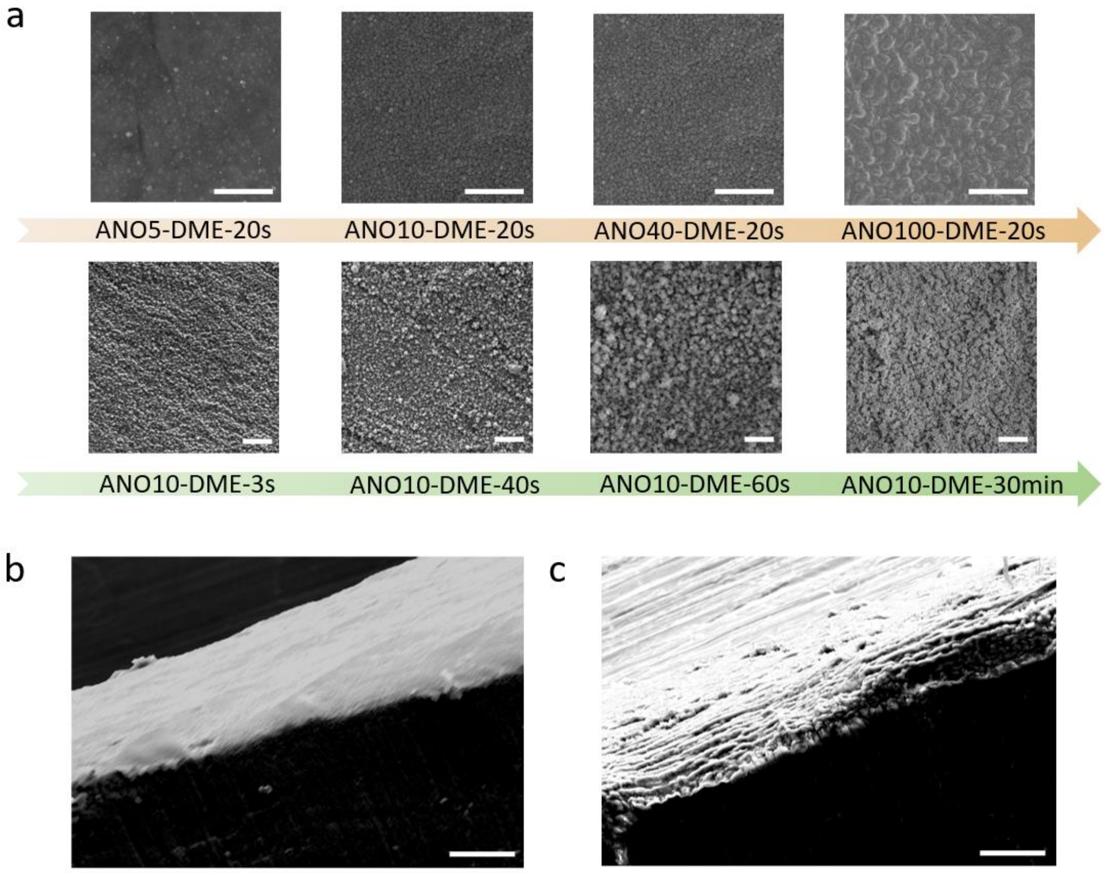

Figure S4. (a) Top-view SEM images of the ANO-DME-Li anodes with different concentrations or reaction times (Scale bars are 10 μm). Cross-sectional SEM images of (b) the ANO10-DME-Li-20s anode and (c) the ANO10-DME-Li-30min anode (Scale bars are 40 μm). The ANO10-DME-Li-20s and ANO40-DME-Li-20s anodes show similar uniform morphology with a little rough and densely packed nanoparticles distributed on their surfaces. The thickness of the alloy layer for ANO*x*-DME-Li varies from 5-10 µm depending on the reaction concentration or time. Considering the sufficient free lithium underneath the alloy layer (~10 µm *vs.* 500 µm), the alloy layer can maintain a consistent composition of the alloy phase through Li^0 self-diffusion, as the Li-rich alloy phase is more thermodynamically stable.[4]

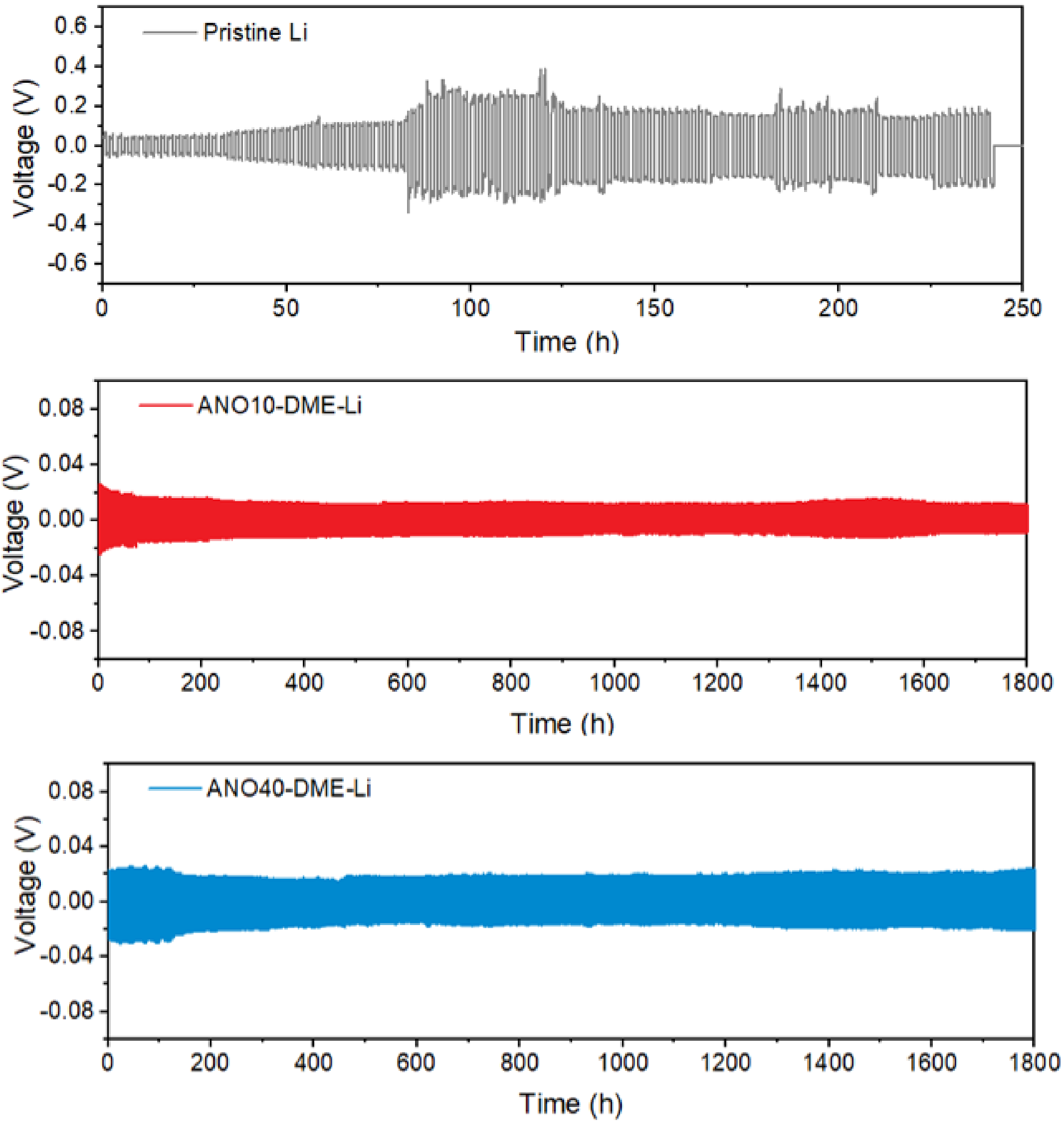

Figure S5. Voltage profiles of LE symmetric cells with pristine Li, ANO10-DME-Li, and ANO40-DME-Li at 1 mA cm^{-2} with a capacity of 1 mAh cm^{-2}.

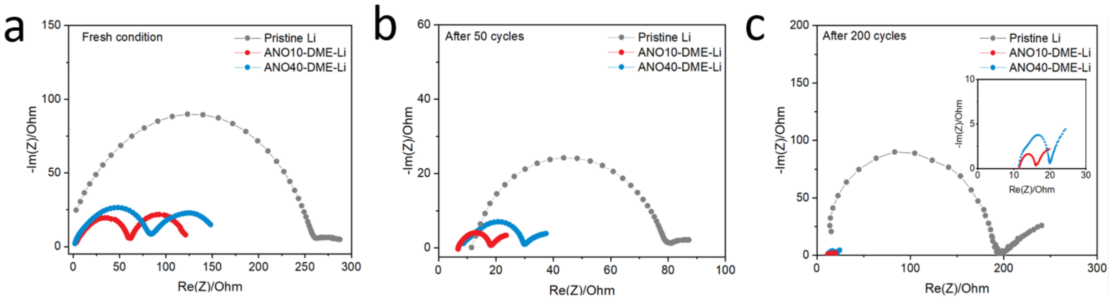

Figure S6. Nyquist plots of symmetric cells with pristine Li, ANO10-DME-Li, and ANO40-DME-Li (a) at fresh conditions, (b) after 50 cycles, and (c) after 200 cycles at 1 mA cm^{-2}.

As shown in Figure S6a, the single semicircle in the Nyquist plot of pristine Li indicates large charge transfer resistance (R_{ct}) between the pristine Li anode and the LE. In contrast, the ANO10-DME-Li and ANO40-DME-Li show two semicircles. The first semicircle in the high-frequency range indicates the interfacial resistance of the interlayer or resistance of Li^+ flux through an interlayer, and the second semicircle in the low-frequency range indicates the R_{ct} between the CRI-alloy interlayer and the LE. The lower R_{ct} of the CRI-alloy-protected anodes before and after cycling can be attributed to the effective control of lithium plating/stripping. While the higher resistances of symmetric cells before plating/stripping cycling can be attributed to the lower electrolyte wettability of the Li electrode that leads to sluggish Li-ion transport.[5] In this regard, the CRI-alloy-protected anode exhibits better wettability towards liquid electrolytes than the pristine anode.

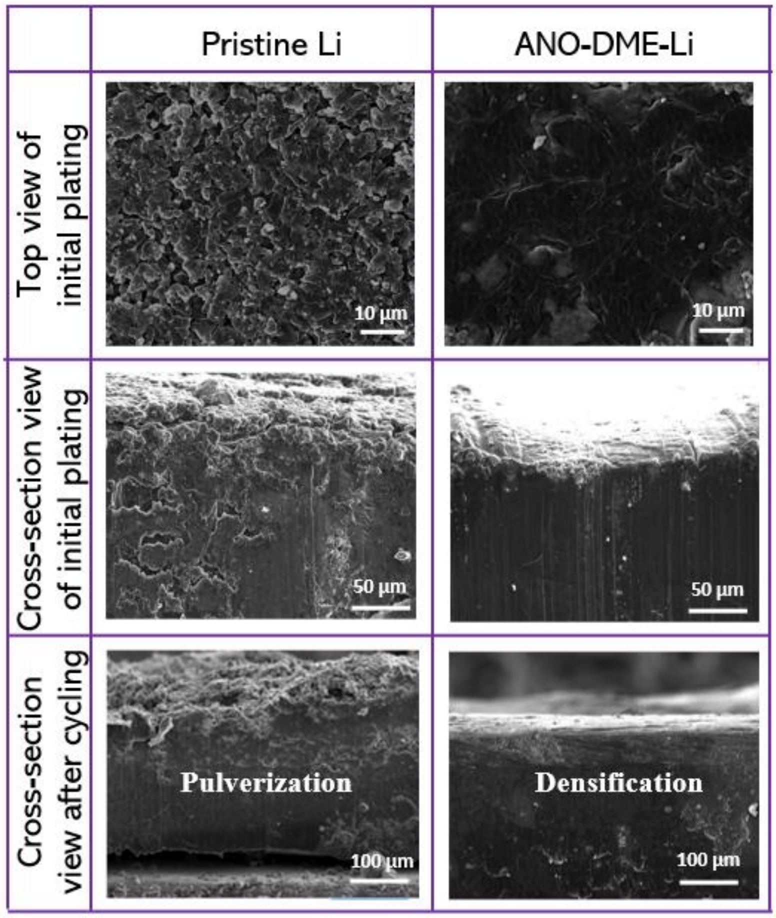

Figure S7. SEM images of lithium deposition morphologies after initial plating and after cycle completion in the LE symmetric cells.

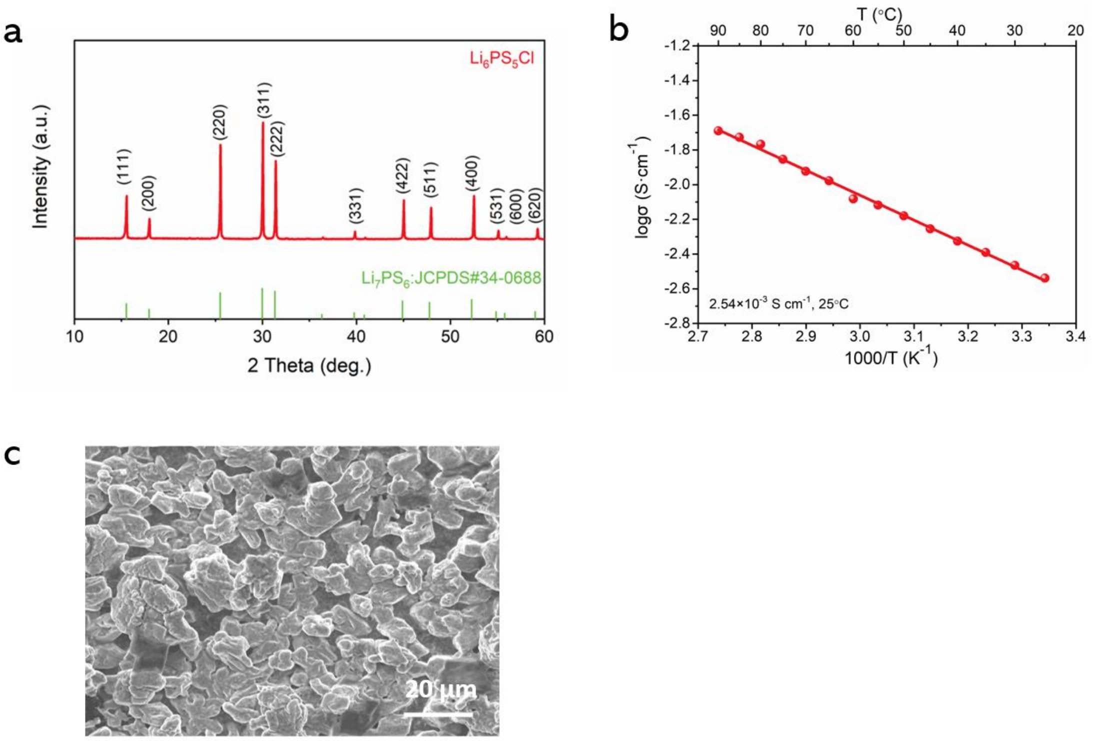

Figure S8. (a) XRD pattern, (b) Arrhenius plots of ion conductivity, and (c) SEM image for the prepared Li_6PS_5Cl SE.

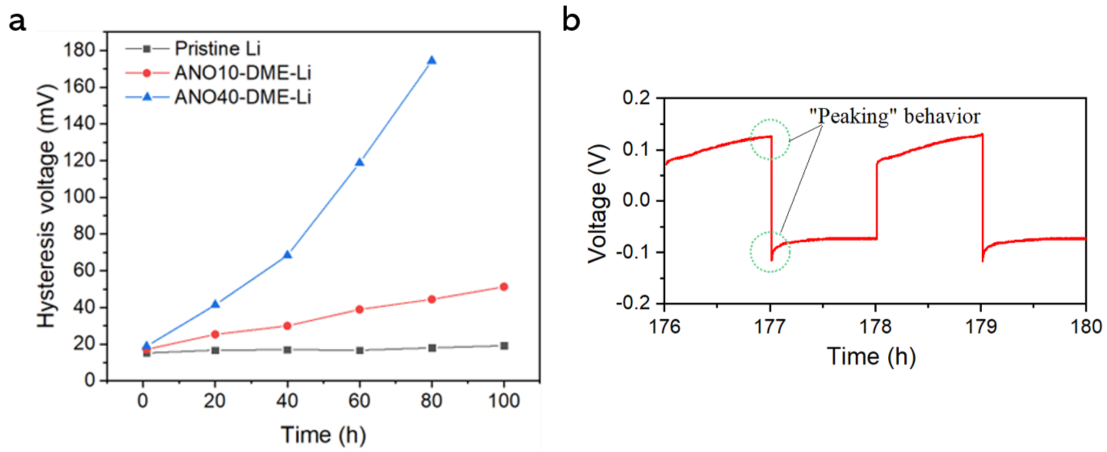

Figure S9. (a) The corresponding average voltage hysteresis from Figure 1a-c. (b) The typical “peaking behavior” presented in voltage traces of SE symmetric cells employing the CRI-alloy-protected Li anodes.

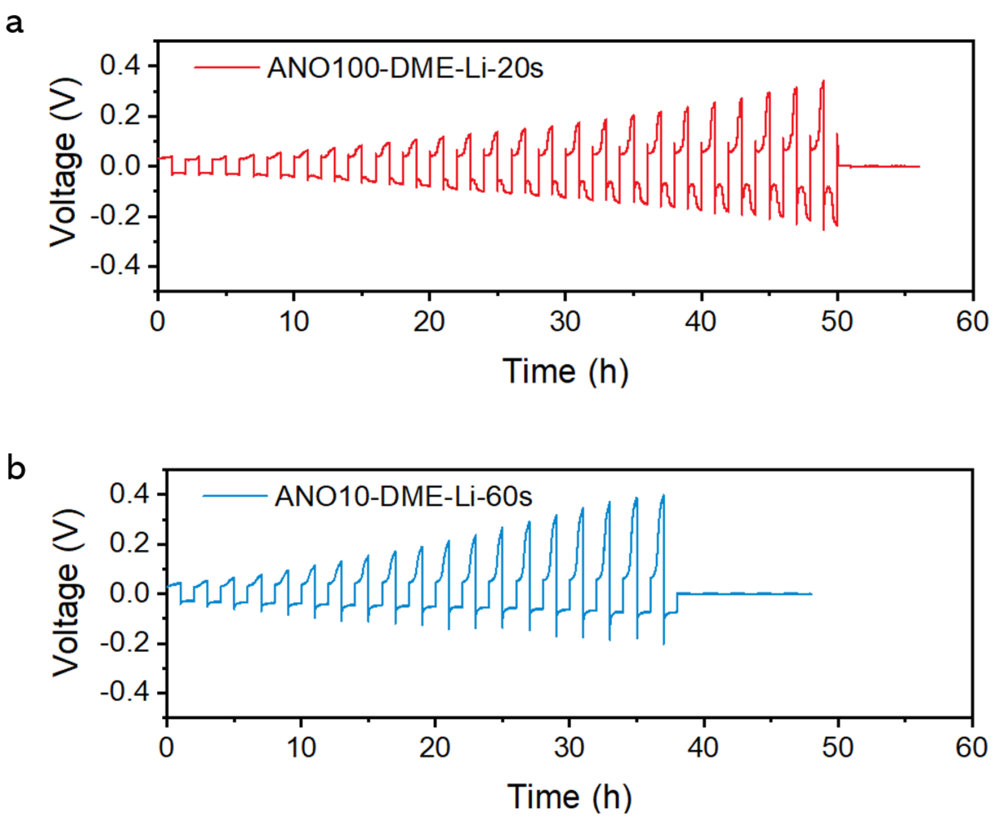

Figure S10. Typical plating/stripping voltage profile of SE symmetric cells employing (a) ANO100-DME-Li-20s and (b) ANO10-DME-Li-60s at 0.1 1 mA cm^{-2}.

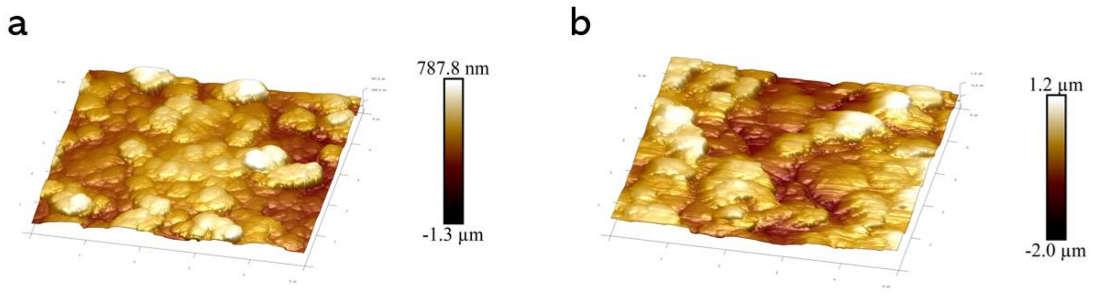

Figure S11. 3D AFM topography images of (a) ANO10-DME-Li and (b) ANO40-DME-Li.

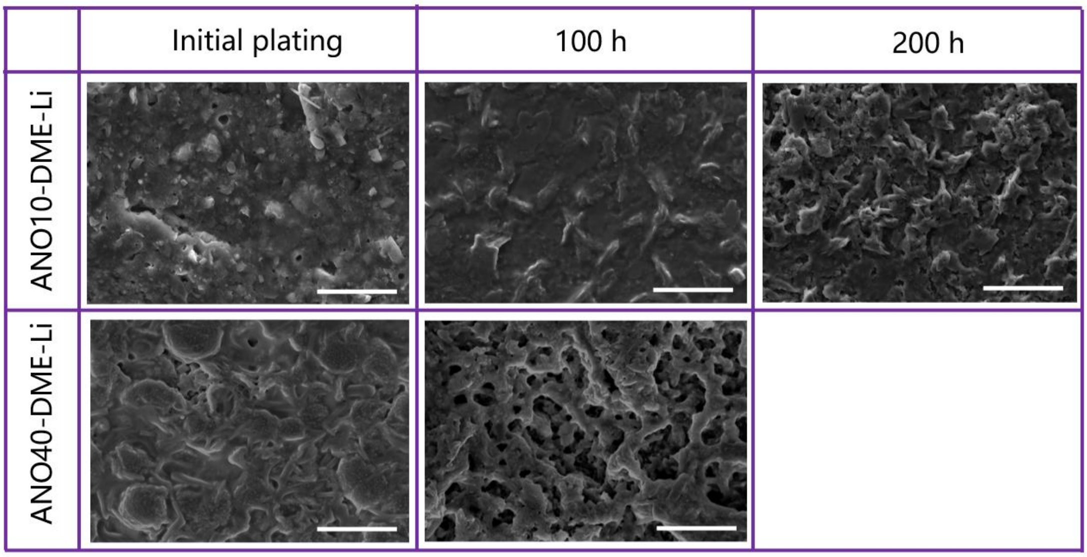

Figure S12. SEM images of lithium deposition morphologies with the CRI-alloy interlayers during cycling in SE symmetric cells. Scale bars are 10 μm. Considering that the lithium deposition and stripping tests were conducted at a very low current density and areal capacity (0.1 mA cm^{-2} and 0.1 mAh cm^{-2}, respectively), we consider that this phenomenon is not related to the amount of lithium platting/stripping, as it would be exacerbated at higher currents. Similarly, it is not strongly correlated with the thickness of the alloy layer, as it is essentially an epitaxial growth case.

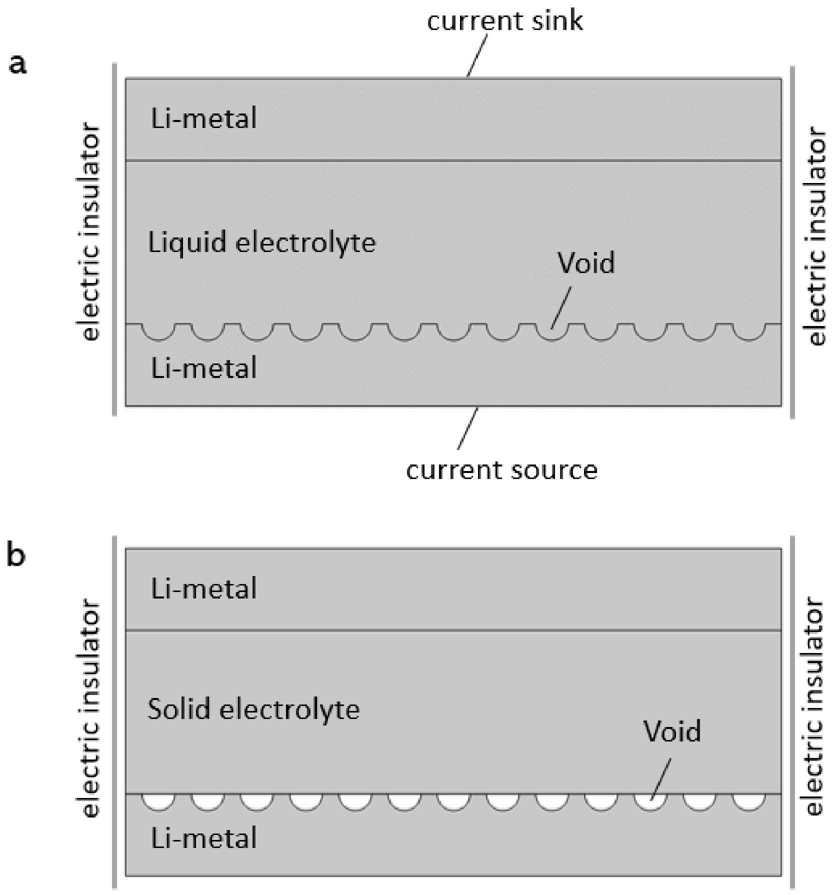

Figure S13. Geometry used for modelling crrent density distribution in the vicinity of voids in (a) LE system and (b) SE system. In the simulation, the external surface of the bottom lithium electrode is connected to a current source and the external surface of the top lithium electrode is connected to a current sink (ground). The other surfaces are set as electrically insulating boundaries. Semicircles (4 μm in diameter) were used to simulate voids at the interface between the Li anode and the SE separator. The *x*-axis is chosen to be along the direction of the interface between the Li electrode and the SE separator. The *y*-axis is chosen to be along the normal to the interface between he Li electrode and the SE separator.

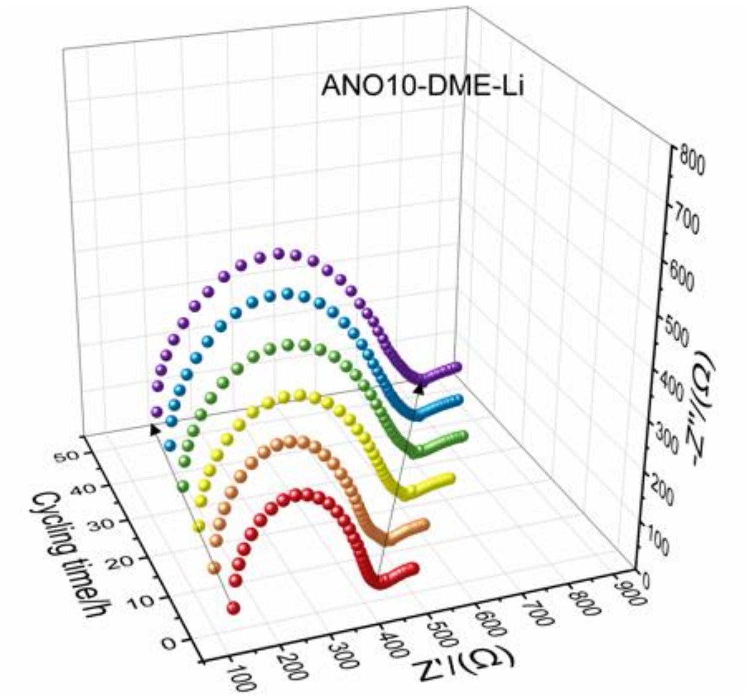

Figure S14. Time-dependent EIS spectra over 50 h of symmetrical ANO10-DME-Li cells at the current density of 0.1 mA cm^{-2} in the SE system. The ever-increasing bulk SE and charge transfer resistance indicate continuous degradation of SE at the SE/Li^0 interface.

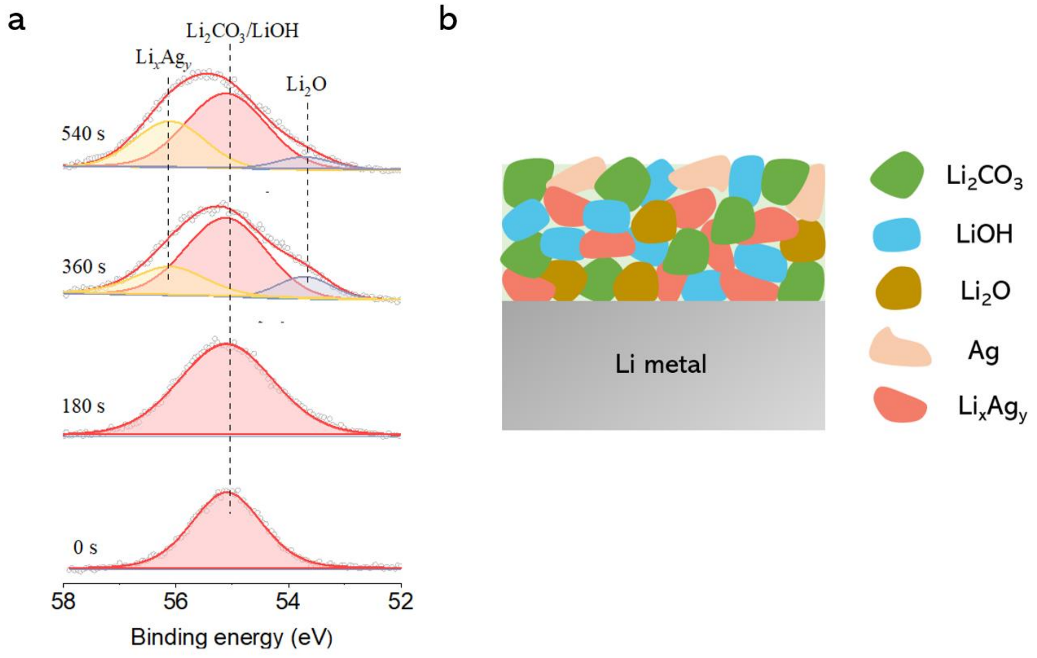

Figure S15. (a) High-resolution XPS depth profiles of Li 1s of ANO10-DME-Li samples. (b) Schematic illustration of the inorganic mosaic structure formed on the ANO-DME-Li anode.

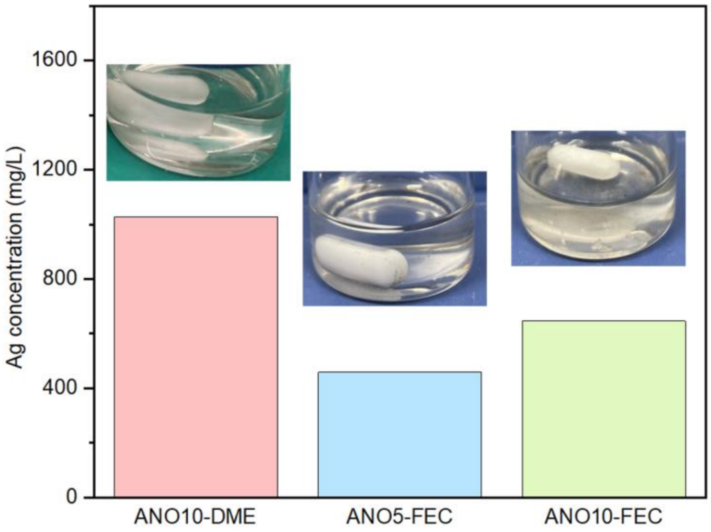

Figure S16. Ag concentrations in ANO10-DME, ANO5-FEC, and ANO10-FEC solutions measured by ICP. Insets are the corresponding photographs. In ANO10-DME and ANO5-FEC solutions, the detectable Ag concentration is 9.5 and 4.2 mM, respectively, which indicates that the dynamic dissociation-recombination equilibrium of $AgNO_3$ was established in these solutions. In contrast, the Ag concentration (5.9 mM) in the cloudy ANO10-FEC solution deviates from its theoretical value implying that the solution is saturated with $AgNO_3$. Therefore, we used 5 mM of $AgNO_3$ dissolved in FEC as the preferred concentration condition in the latter investigation.

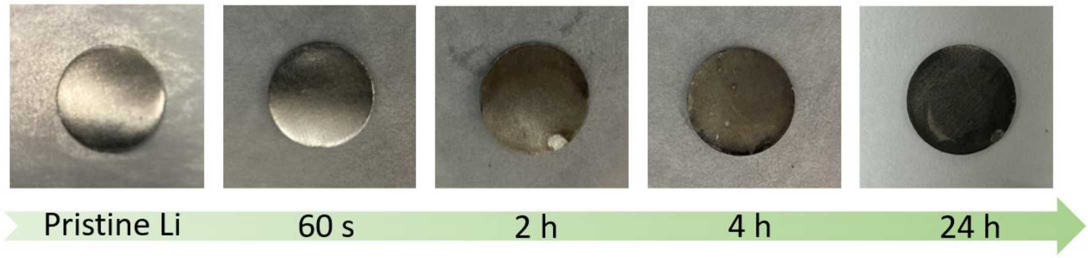

Figure S17. Optical photographs of Li anodes after immersion in ANO-FEC solution for different times. Seconds of immersion time cannot effectively promote the discoloration of Li foil. The extended time required for electrode discoloration to occur implies a different reaction mechanism in the ANO-FEC case.

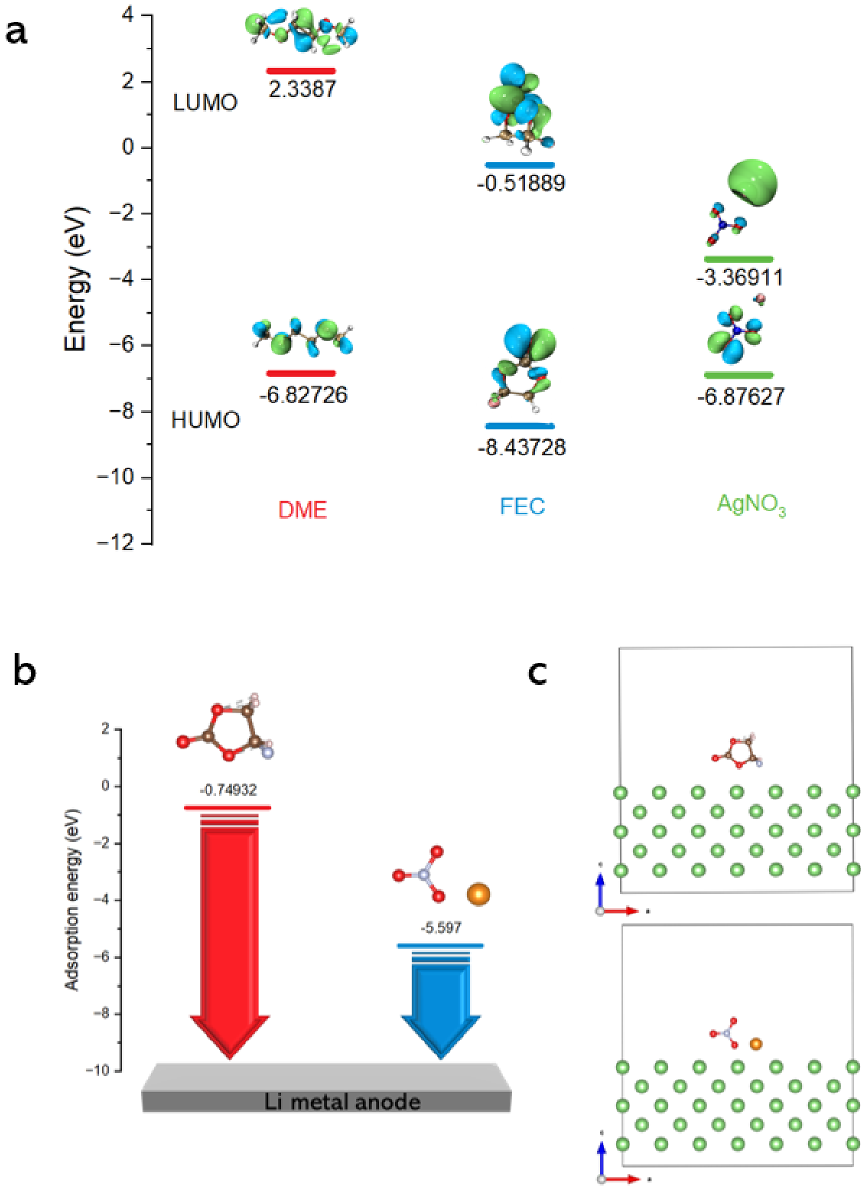

Figure S18. (a) HOMO and LUMO levels of DME, FEC, and $AgNO_3$. (b) Schematic illustration of adsorption energies of FEC and $AgNO_3$ on the Li(100) surface. (c) Adsorption models for calculating adsorption energies of FEC and $AgNO_3$ on the Li(100) surface.

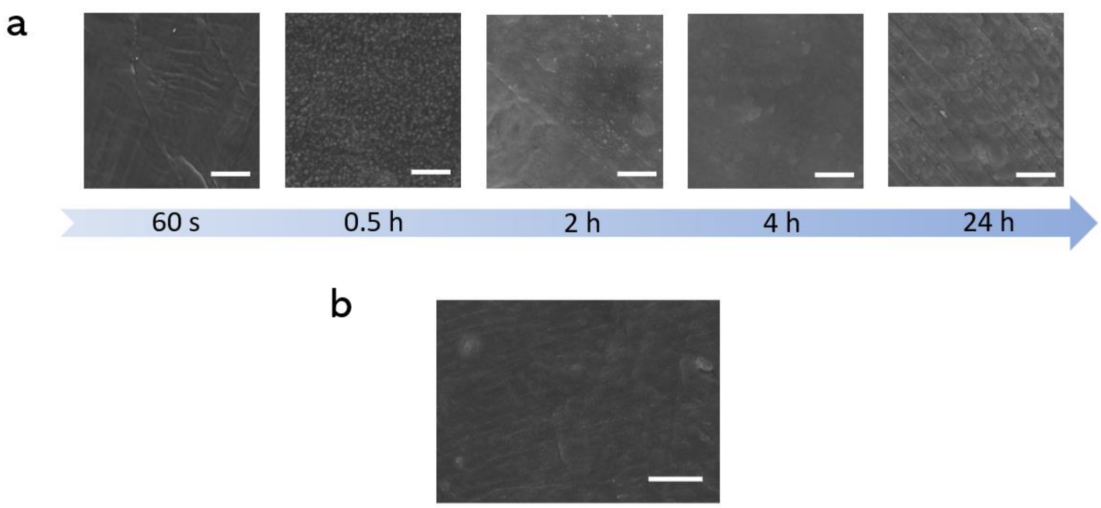

Figure S19. (a) SEM images of ANO-FEC-Li anodes with varying soaking times. (b) SEM image of the Li anode immersed in neat FEC solvent for 4 h. Scale bars are 10 μm.

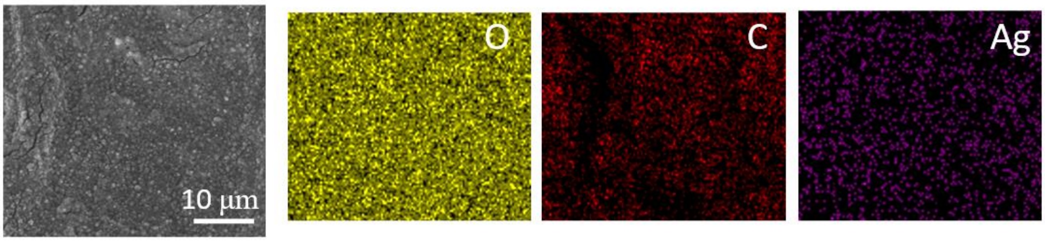

Figure S20. SEM image of the ANO-DME-Li anode and the corresponding EDS maps of O, C, and Ag.

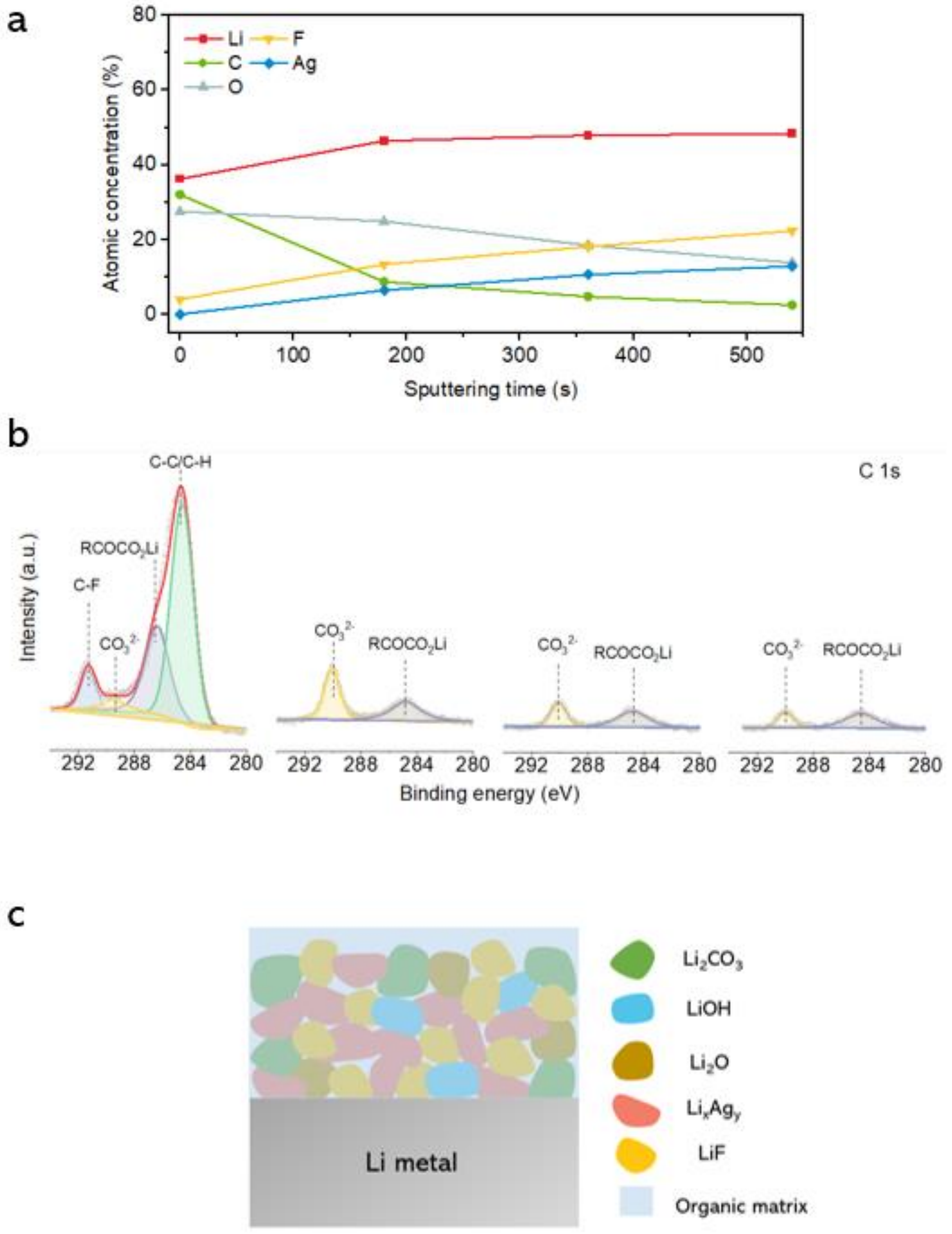

Figure S21. (a) The evolution profiles of atomic percentages along the depth directions. (b) High-resolution XPS depth profiles of C1s of the ANO-FEC-Li anode. (c) Schematic illustration of the organic-inorganic hierarchical gradient structure formed on the ANO-FEC-Li anode.

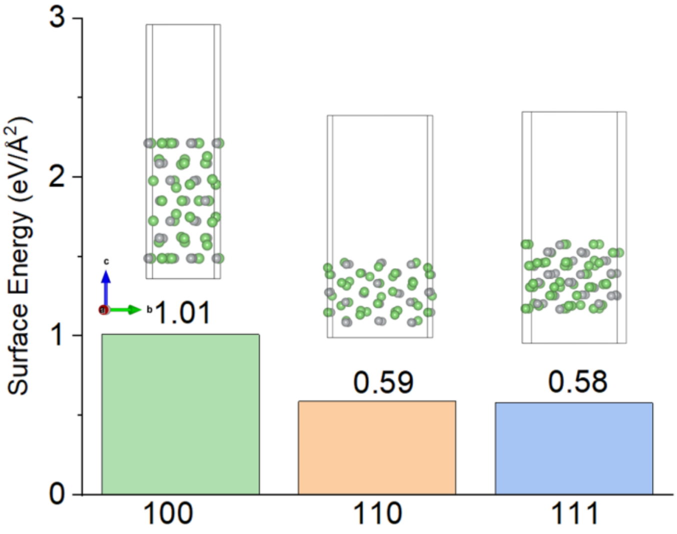

Figure S22. Absorption sites and the corresponding surface energy for Li on the Li_8Ag_5 (110) (110) and (111) surfaces. The corresponding surface energies are 1.01 eV/Å^2, 0.59 eV/Å^2, and 0.58 eV/Å^2, respectively. Therefore, Li_8Ag_5 (111) is the most stable surface.

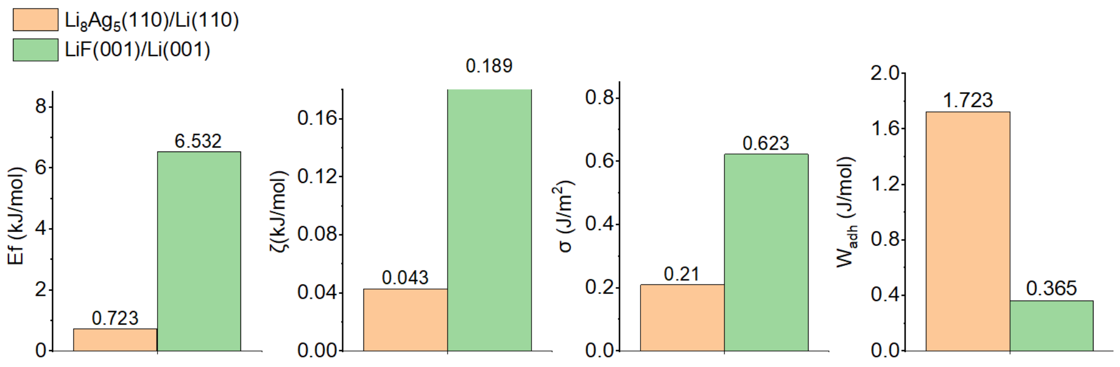

Figure S23. The calculated formation energy (E_f), strain energy (ζ), interfacial energy (σ), and the work of adhesion (W_{adh}) for the interfacial supercells.

The interface properties including the formation energy (E_f), strain energy (ζ), interfacial energy (σ), and the work of adhesion (W_{adh}) of the interlayer were calculated based on previous studies.[6,7]

The formation energy (E_f) of an interfacial phase can be determined by calculating the difference in energy between the relaxed interfacial phase and two stress-free pure phases with the same atomic number or minimum number of elements. If the interfacial phase is composed of AB (LiF or Li_8Ag_5) and Li metal, the formation energy can be calculated using the formula:

$$E_f = E_{AB/Li} - E_{AB}^{AB-bulk} - E_{Li}^{Li-bulk}$$

Here, $E_{AB/Li}$ is the energy of the fully relaxed interfacial supercell model, while $E_{AB}^{AB-bulk}$ and $E_{Li}^{Li-bulk}$ are the energies of the AB and the Li part, respectively, under the state of no stress.

The interfacial energy (σ) can be calculated using the following formula:

$$\sigma = [E_{AB/Li} - E_{AB}^{AB-bulk(c)} - E_{Li}^{Li-bulk(c)}]/2\mathrm{A}$$

where $E_{AB}^{AB-bulk(c)}$ and $E_{Li}^{Li-bulk(c)}$ are the energies the constrained bulks of AB and Li, respectively. Only axis C is relaxed while axis A and B are not relaxed.

The work of adhension (W_{adh}) can be calculated using the following formula:

$$W_{adh} = \gamma_{AB} + \gamma_{Li} - \sigma_{AB/Li}$$

where γ_{AB} and γ_{Li} are the surface energies of AB and Li in the interface model, respectively.

The strain energy ζ is calculated following formula:

$$\frac{E_f}{V} = \frac{2A\sigma}{V} + \zeta$$

where V represents the volume of the interface model in the fully relaxed state.

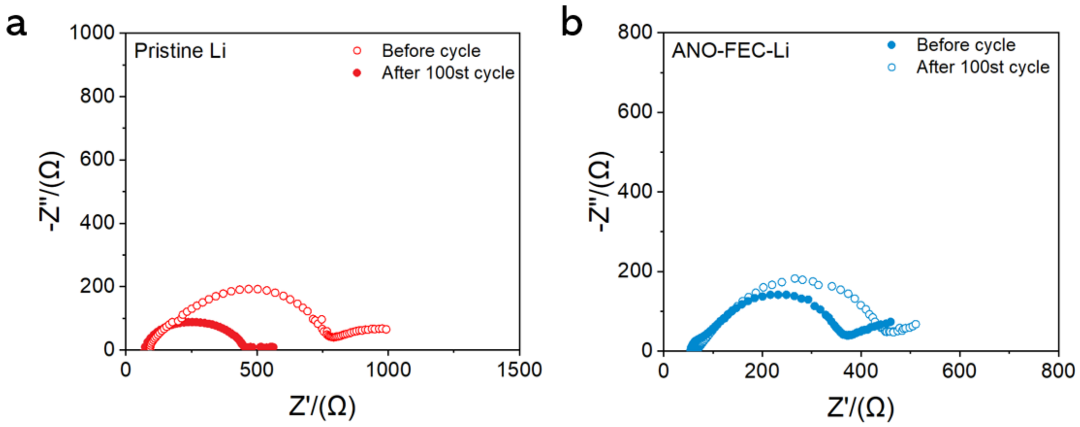

Figure S24. Evolution of the electrochemical impedance spectroscopy spectra for (a) pristine Li and (b) ANO-FEC-Li anodes in symmetric cells.

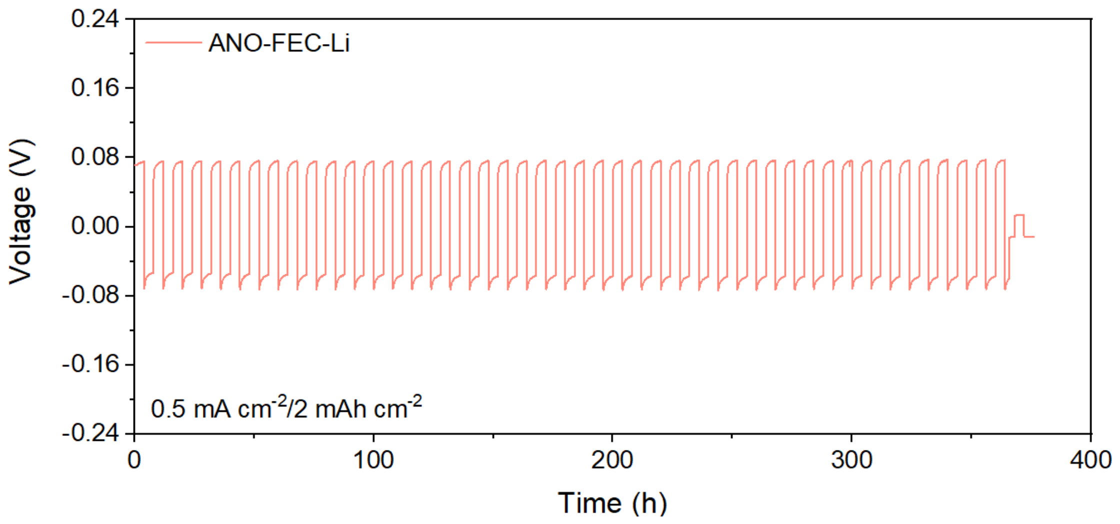

Figure S25. Galvanostatic Li plating/stripping profiles of symmetric cells with ANO-FEC-Li at the current density of 0.5 mA cm^{-2} with an areal capacity of 2 mAh cm^{-2}.

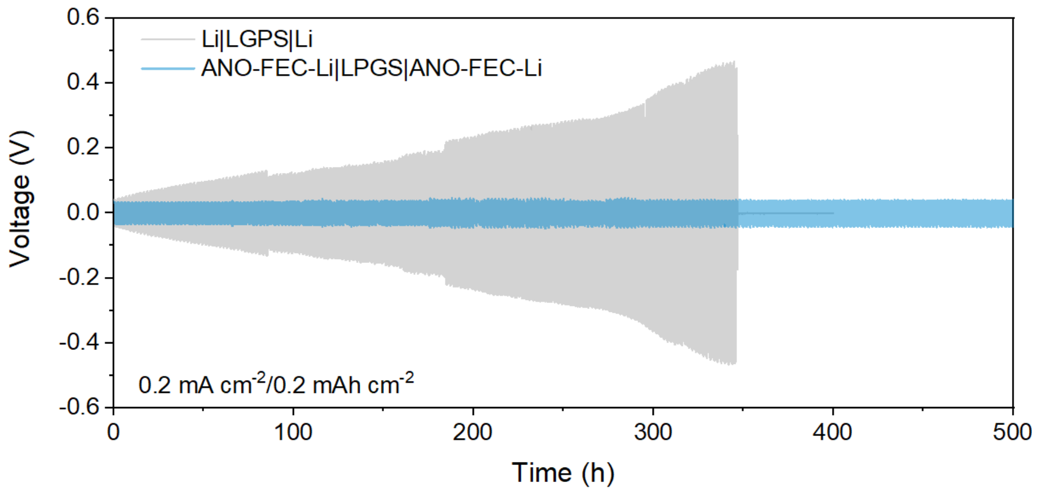

Figure S26. Galvanostatic Li plating/stripping profiles of LGPS-based symmetric cells with pristine Li and ANO-FEC-Li at the current density of 0.2 mA cm^{-2} with an areal capacity of 0.2 mAh cm^{-2}.

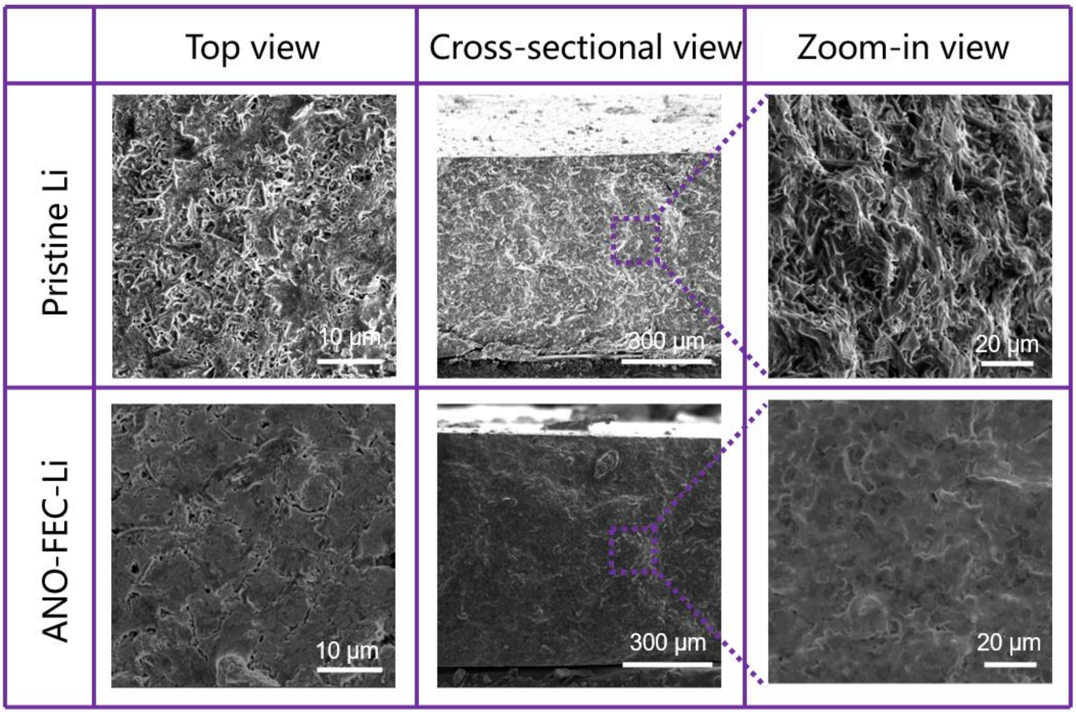

Figure S27. SEM images of SE separators paired with pristine Li and ANO-FEC-Li anodes in symmetric cells after cycling at 0.5 mA cm^{-2}.

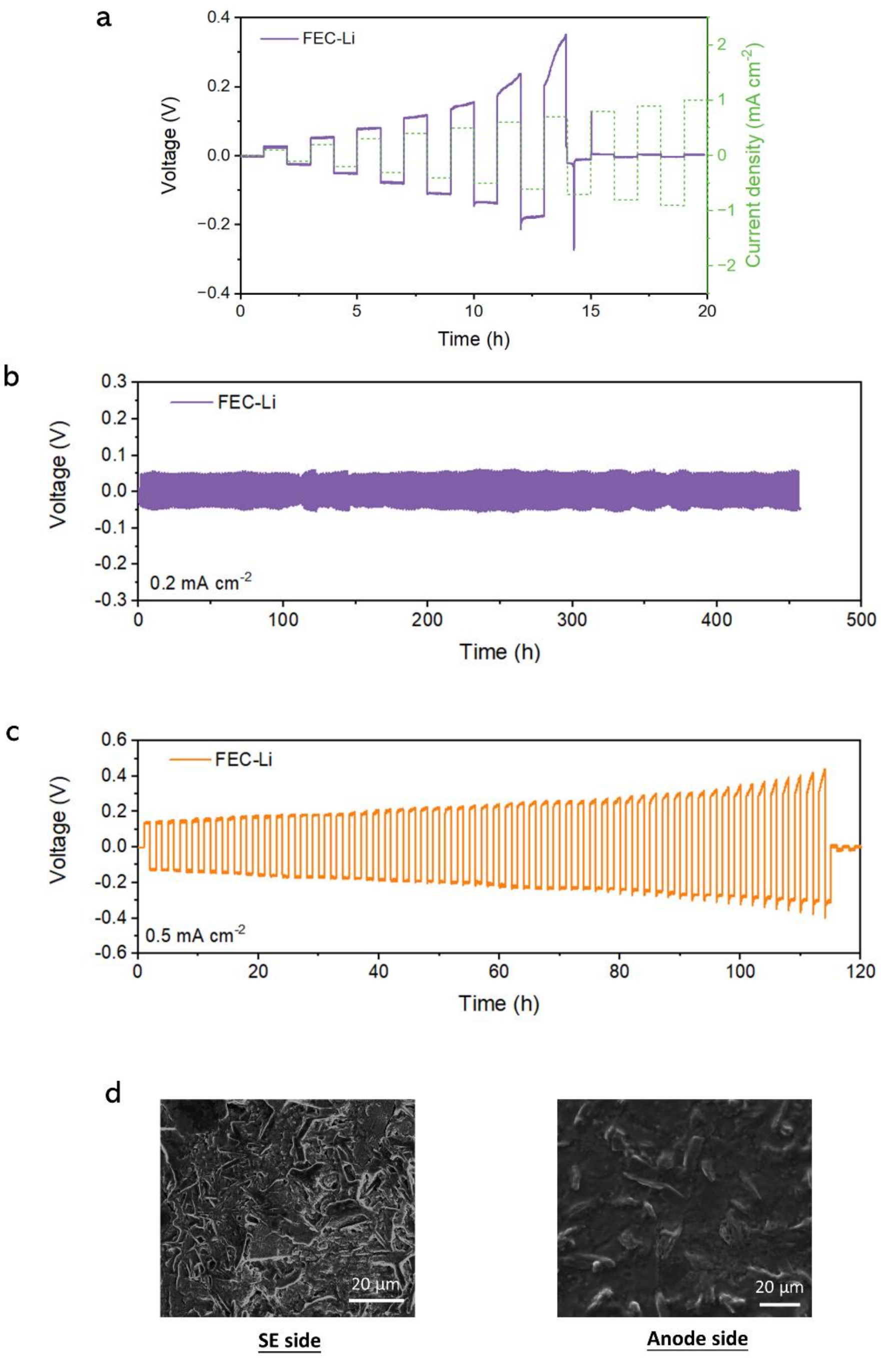

Figure S28. (a) Galvanostatic cycling of FEC-Li symmetric cells under step-increased current densities of 0.1 mA cm^{-2} with a constant half-cycle duration of 1 h. Galvanostatic Li plating/stripping profiles of FEC-Li symmetric cells at the current density of (b) 0.2 mA cm^{-2} and (c) 0.5 mA cm^{-2}. (d) Top-view SEM images of SE and FEC-Li anode after galvanostatic Li plating/stripping tests at 0.5 mA cm^{-2}.

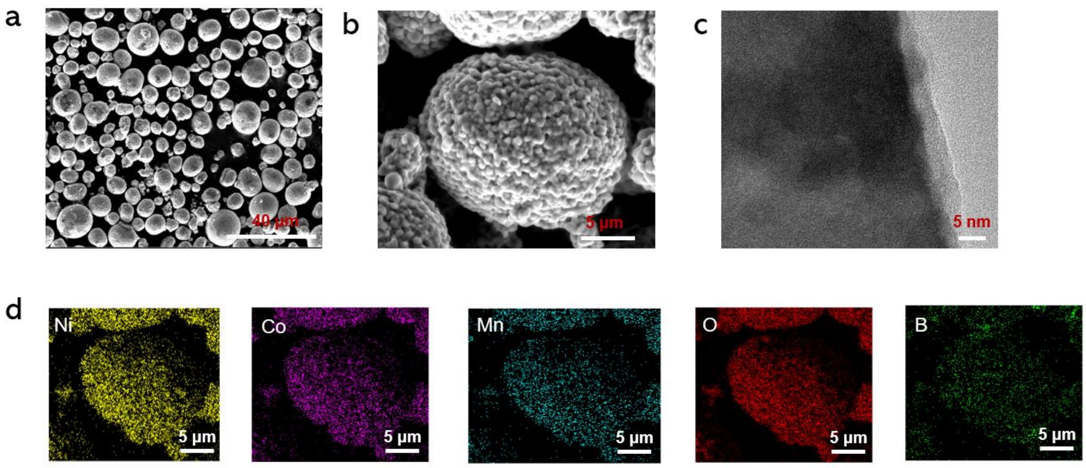

Figure S29. (a) Low- and (b) high-magnification of SEM images for Li_3BO_7-coated $Li(Ni_{0.6}Co_{0.2}Mn_{0.2})O_2$ particles. (c) The high-resolution transmission electron microscopy (HRTEM) image of the Li_3BO_7-coated $Li(Ni_{0.6}Co_{0.2}Mn_{0.2})O_2$ particle. The coating thickness is about 5 nm. (d) The EDS maps of Ni, Co, Mn, O, and B from Fig. S27b. The Li_3BO_7-coated $Li(Ni_{0.6}Co_{0.2}Mn_{0.2})O_2$ was prepared via the sol–gel method as previously reported.

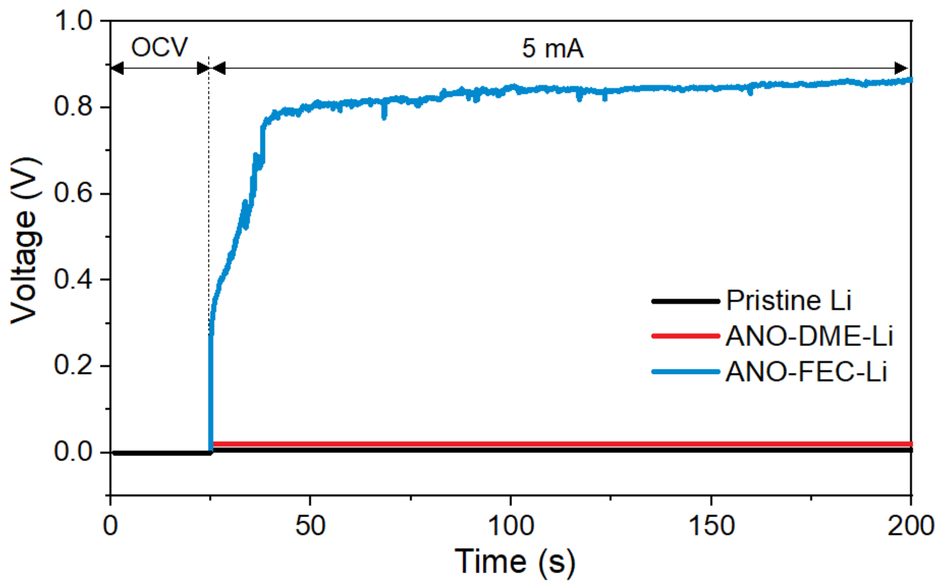

Figure S30. Measurements of d.c. conductivity of pristine Li, ANO-DME-Li, and ANO-FEC-Li anodes using blocking electrodes.

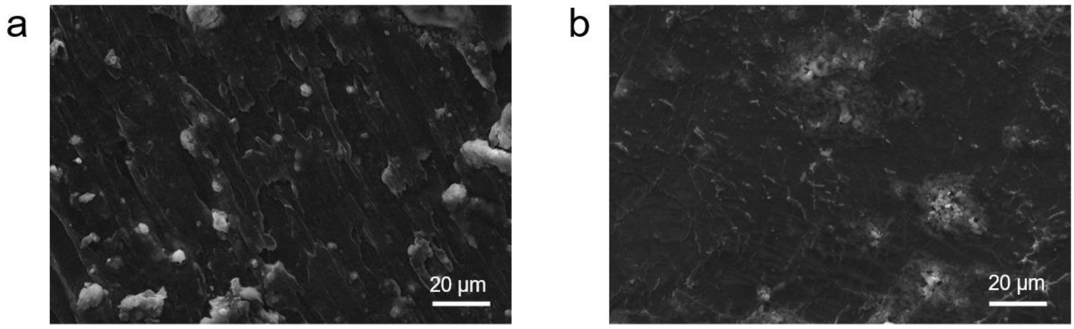

Figure S31. SEM images of (a) pristine Li and (b) ANO-FEC-Li anodes after initial plating with an areal capacity of 2 mAh cm^{-2}.

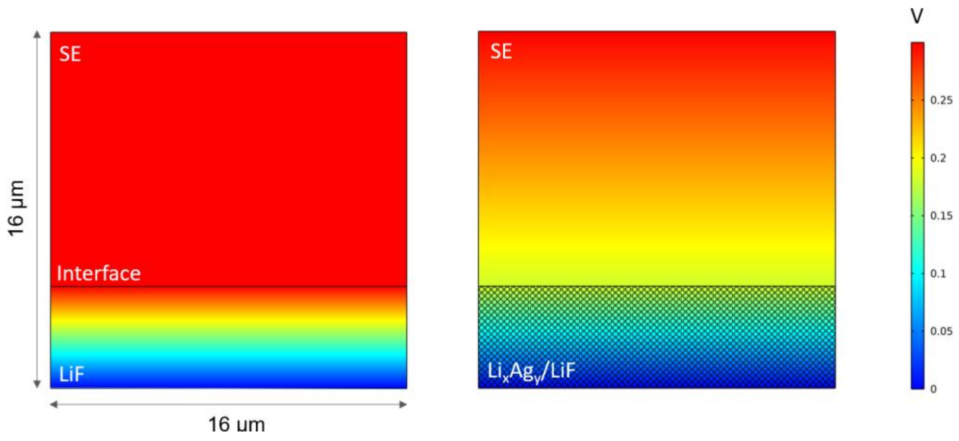

Figure S32. Finite element simulations of potential distribution for the the LiF and Li_xA_g/LiF interlayers under a pre-set overpotential of 0.3 V.

References

[1] G. Kresse, J. Furthmüller, ***Phys. Rev. B*** 1996, 54, 11169.

[2] G. Kresse, D. Joubert, ***Phys. Rev. B*** 1999, 59, 1758.

[3] J. P. Perdew, A. Ruzsinszky, G. I. Csonka, O. A. Vydrov, G. E. Scuseria, L. A. Constantin, X. Zhou, K. Burke, ***Phys. Rev. Lett.*** 2008, 100, 136406.

[4] X. Liang, Q. Pang, I. R. Kochetkov, M. S. Sempere, H. Huang, X. Sun, L. F. Nazar, *Nat. Energy* 2017, 2, 1.

[5] R. Pathak, K. Chen, A. Gurung, K. M. Reza, B. Bahrami, J. Pokharel, A. Baniya, W. He, F. Wu, Y. Zhou, K. Xu, Q. Q. Qiao, *Nat. Commun.* 2020, 11, 93.

[6] A. Hu, W. Chen, X. Du, Y. Hu, T. Lei, H. Wang, L. Xue, Y. Li, H. Sun, Y. Yan, J. Long, C. Shu, J. Zhu, B. Li, X. Wang, J. Xiong, *Energy Environ. Sci*. 2021. 14, 4115-4124.

[7] W. Li, J. Gao, H. Tian, X. Li, S. He, J. Li, W. Wang, L. Li, H. Li, J. Qiu, W. Zhou, *Angew. Chem. Int. Ed. Engl.* 2021, 61, e202114805.
